# Working across religions, cultures, settings, and development: Protocol for wave 2 data collection with children and parents by the developing belief network

**DOI:** 10.1371/journal.pone.0330727

**Published:** 2025-09-05

**Authors:** Allison J. Williams-Gant, Kara Weisman, Tamer G. Amin, Maliki E. Ghossainy, Ghadir Soueidan, Jenny Nissel, Praveen Kenderla, Marwa Abdel-Hak, Florencia K. Anggoro, Samantha Bangayan, Emily R. R. Burdett, Emily Chau, Eva E. Chen, Jallene Chua, Lezanie Coetzee, John D. Coley, Audun Dahl, Jocelyn B. Dautel, Elizabeth L. Davis, Helen Elizabeth Davis, Adine DeLeon, Gil Diesendruck, Denise Evans, Aidan Feeney, Frankie T. K. Fong, Xuqing Foo, Alison Garcia, Isabela Gonzalez-Rubio, Elena Guerrero Galaz, Michael Gurven, Ying Hu, Keila Huachorunto, Komang Indrawati, Benjamin D. Jee, Michael Kahwa, Unity Kahwa, Ringking Korah, Hannah J. Kramer, Tamar Kushnir, Natassa Kyriakopoulou, Shitshembiso Lebepe, Hea Jung Lee, Kirsten A. Lesage, Patricia Leshabana, Dandan Li, Pearl Han Li, Jessica Tacza Llacua, Vongani Maluleke, Ashley Marin, Julia Marshall, Nthabiseng Masebe, Katherine McAuliffe, Abby McLaughlin, Anthea McMullan, Caitlin McShane, Casey Min, Mike Mutegeki, Olive Namara, Shaun Nichols, Ageliki Nicolopoulou, Mark Nielsen, Emily Otali, Katerina Parise, Xiomara Alicia Paucar, Ayse Payir, Sakina Poonawalla, Bolivar Reyes-Jaquez, Sophie Riddick, Peter C. Rockers, Justin K. Ruiz, Rifah Sanjidah, Laura Shneidman, Irini Skopeliti, Mahesh Srinivasan, Jessa Stegall, Joanna Stephens, Megan G. Stutesman, Jiayue Sun, Amanda Tarullo, Laura K. Taylor, Itangishatse Theogen, Desiree Toong, Esra Nur Turan-Küçük, Patrick Tusiime, Estefany Pizarro Ventura, Jingyi Xu, Nina Ye, Yue Yu, Meltem Yucel, Wenzhuo Zhang, Xin Zhao, Kathleen H. Corriveau, Rebekah A. Richert, on behalf of the Developing Belief Network

**Affiliations:** 1 Wheelock College of Education & Human Development, Boston University, Boston, Massachusetts, United States of America; 2 Department of Psychology, University of California, Riverside, California, United States of America; 3 Department of Education, American University of Beirut, Beirut, Lebanon; 4 Department of Language Sciences and Traductology, Holy Spirit University of Kaslik, Jounieh, Lebanon; 5 Department of Psychology, College of the Holy Cross, Worcester, Massachusetts, United States of America; 6 Department of Psychology and Neuroscience, Boston College, Chestnut Hill, Massachusetts, United States of America; 7 School of Psychology, University of Nottingham, Nottingham, United Kingdom; 8 Department of Psychology, University of California, Berkeley, California, United States of America; 9 College of Education, National Tsing Hua University, Hsinchu, Taiwan, Republic of China; 10 Singapore Centre for Character and Citizenship Education, National Institute of Education, Nanyang Technological University, Singapore; 11 Health Economics and Epidemiology Research Office (HE2RO), University of the Witwatersrand, Johannesburg, South Africa; 12 Department of Psychology, Northeastern University, Boston, Massachusetts, United States of America; 13 Department of Marine & Environmental Sciences, Northeastern University, Boston, United States of America; 14 Department of Psychology, Cornell University, Ithaca, New York, United States of America; 15 School of Psychology, Queen’s University Belfast, Belfast, United Kingdom; 16 School of Human Evolution and Social Change, Arizona State University, Tempe, Arizona, United States of America; 17 Department of Psychology, Bar-Ilan University, Ramat-Gan, Israel; 18 Gonda Brain Research Center, Bar-Ilan University, Ramat-Gan, Israel; 19 School of Psychological Sciences, Victoria University of Wellington, Kelburn Campus, Wellington, New Zealand; 20 School of Medical and Life Sciences, Sunway University, Bandar Sunway, Selangor, Malaysia; 21 School of Psychology, University of Queensland, St Lucia Campus, Brisbane, Australia; 22 Department of Anthropology, University of California, Santa Barbara, California, United States of America; 23 Department of Educational Psychology, East China Normal University, Shanghai, Mainland China; 24 I/O Psychology and Educational Psychology, Udayana University, Bali, Indonesia; 25 Department of Psychology, Worcester State University, Worcester, Massachusetts, United States of America; 26 Kibale Forest Schools Program, Kabarole, Uganda; 27 People and Change (PnC) Consulting, Medan, Indonesia; 28 Department of Psychology, University of Wisconsin, Madison, Wisconsin, United States of America; 29 Department of Psychology & Neuroscience, Duke University, Durham, North Carolina, United States of America; 30 Department of Philosophy, Duke University, Durham, North Carolina, United States of America; 31 Department of Early Childhood Education, National and Kapodistrian University of Athens, Athens, Greece; 32 Department of Psychology, University of Exeter, Exeter, United Kingdom; 33 Department of Cognitive and Psychological Sciences, Brown University, Providence, Rhode Island, United States of America; 34 Department of Philosophy, Cornell University, Ithaca, New York, United States of America; 35 Psychology Department, Lehigh University, Bethlehem, Palestinian, United States of America; 36 Department of Communication and Media, University of Johannesburg, Auckland Park, South Africa; 37 Department of Early Childhood Education, University of Patras, Patras, Greece; 38 Department of Psychology, Union College, Schenectady, New York, United States of America; 39 Department of Psychology, College of Liberal Arts, University of New Hampshire, Durham, New Hampshire, United States of America; 40 Boston University School of Public Health, Boston University, Boston, Massachusetts, United States of America; 41 Department of Psychology, Pacific Lutheran University, Tacoma, Washington, United States of America; 42 Department of Philosophy and History of Science, National and Kapodistrian University of Athens, Athens, Greece; 43 Department of Psychological & Brain Sciences, Boston University, Boston, Massachusetts, United States of America; 44 School of Psychology, University College Dublin, Ireland; 45 Centre for Research in Child Development, National Institute of Education, Nanyang Technological University, Singapore; PLOS: Public Library of Science, UNITED KINGDOM OF GREAT BRITAIN AND NORTHERN IRELAND

## Abstract

The Developing Belief Network is a global research collaborative studying religious development in diverse social-cultural settings, with a focus on the intersection of cognitive mechanisms and cultural beliefs and practices in early and middle childhood. The current manuscript describes the study protocol for the network’s second wave of data collection, which aims to further explore the development and diversity of religious cognition and behavior using a multi-time point approach. This protocol is designed to investigate three key research questions—how children represent and reason about religious and supernatural agents, how children represent and reason about religion as an aspect of social identity, and how religious and supernatural beliefs are transmitted within and between generations—via a set of eight tasks for children between the ages of 5 and 13 years and a survey completed by their parents/caregivers. This study is being conducted in 41 distinct cultural-religious settings, spanning 16 countries and 12 written languages. In this manuscript, we provide detailed descriptions of all elements of this study protocol, and give a brief overview of the ways in which this protocol has been adapted for use in diverse religious communities. As one example of how this protocol has been implemented outside of the United States, we present Arabic- and English-language study materials for children being raised in one of the following religious traditions in Lebanon: the Druze faith, Maronite Christianity, Orthodox Christianity, Shia Islam, or Sunni Islam. We end with reflections on the challenges of developing and implementing large-scale, multi-site, multi-time point studies of child development; our approach to navigating these challenges; and our suggestions for how future researchers might learn from our experiences and build on the work presented here.

## Introduction

In this paper we describe a study protocol designed to explore the development and diversity of religious cognition and behaviors among 5- to 13-year-old children in a diverse range of cultural and religious settings. This protocol is being used in the second wave of a large-scale, multi-time point study being conducted by the Developing Belief Network (DBN), a consortium of psychological scientists and other scholars working in field sites distributed across the globe. An earlier manuscript described the tasks administered to children and their parents/caregivers at Wave 1 of this study [1]. This paper describes the tasks that are being administered to participants at Wave 2 of this study, and outlines the changes between Waves 1 and 2. In particular, we discuss the need to address three competing goals of our research network: repeating measures across waves of data collection to facilitate longitudinal analyses; refining our measures to be more developmentally appropriate and more culturally sensitive, based on experiences in the field during the first wave of data collection; and shortening the length of the protocol to address the stress on resources for both the research teams and the participating families. We argue that the challenge of striking a balance between these three competing goals is inherent to this style of multi-site, multi-time point, culturally attuned research; we conclude with a meditation for other research teams on how to navigate these challenges.

As described in [1], the DBN is a global research consortium studying religious development in diverse social-cultural settings. The research is guided by a focus on the interplay between general cognitive development and culturally-specific processes of socialization and cultural transmission in early and middle childhood. The DBN consists of 13 research teams, encompassing over 200 individuals who have contributed to our collaborative research. Since the publication of the protocol paper for Wave 1 the DBN has added one research team to the network (PI: Frankie Fong; co-PI: Mark Nielsen; samples: Christians living in Tenom, East Malaysia; Buddhists in Kuala Lumpur, Malaysia; and Christians and religiously unaffiliated families living in Brisbane, Australia), and one research team has added a sample to their field work (PI: Florencia Anggoro; co-PI: Benjamin Jee; new sample: Buddhists living in Jakarta, Indonesia). Across all waves of data collection, the DBN has worked in 46 distinct cultural-religious settings, spanning 19 countries and written 15 languages. At the time of this submission, Wave 2 data collection is underway in 40 cultural-religious samples, spanning 16 countries and 12 written languages (see “Materials and Methods,” below).

Studies of religious development provide unique insights into the interactions between cognitive development, socialization, and enculturation [2], shedding new light on how children in different cultural-religious settings come to think, behave, experience, and relate to the world around them. Since many religious beliefs involve reasoning about entities and phenomena that are difficult (or impossible) to observe, children must rely on a variety of other processes to acquire and refine their understanding of religion, such as cultural and social learning [3, 4], learning through testimony [5], learning through text [6, 7], and learning through participation and observation [8, 9]. The DBN has three major research questions that are addressed by all waves of data collection [1]: How do children represent and reason about religious and supernatural agents? How do children represent and reason about religion as an aspect of social identity? And how are religious and supernatural beliefs transmitted within and between generations?

To answer these research questions, the Wave 1 protocol employed cross-sectional planned analyses. However, one of the primary goals of the original research grant submission was to examine religious development within the lives of individual children, returning to the same participants roughly one and two years later. Adopting this within-subjects, multi-time point approach to exploring religious development allows us to examine how individual children’s cognition evolved over that time. Because learning about religion is both culturally and individually specific, individual differences in religious development are especially important to capture; employing a multi-time point approach allows researchers to statistically control for some of the variance explained by individual factors [10,11]. In addition to the accumulation of religious knowledge over time, children’s understanding of and relation to their religion is often marked by specific moments of dramatic change, such as undergoing the coming-of-age rituals common to many religious traditions, or living through historical events that highlight religious identities or increase the tension between religious groups. Such moments might occur at different times in a child’s life, both within and across faiths and cultural groups. A multi-time point approach can capture such “pre/post” differences in a way that a cross-sectional approach cannot.

We characterize our approach as “within-subjects” and “multi-time point,” but not strictly “longitudinal.” Longitudinal research often includes a research design involving observing or measuring the *same* variables over a set period of time. In this study, we were able to preserve some variables across time points, but there were several important motivations for revisions between Wave 1 and Wave 2: refining our measures to be more developmentally appropriate and more culturally sensitive, and shortening the length of the protocol to address the stress on resources for both the research teams and the participating families.

Turning first to the refinements we made to be more developmentally appropriate and more culturally sensitive, recent research in developmental psychology has emphasized that the existing methods created and used in so-called “WEIRD” [12] or “Minority World” [13] settings may not be valid measures to use with children in other settings [14–16]. To take one prominent example, recent debates on how best to conceptualize and measure the development of executive function have highlighted that many of the tasks previously used in Western countries do not take into account the lived experiences of individuals in other social-cultural settings [14]. Answering correctly in a Day/Night Stroop task requires saying something that might be construed as “untrue” (for example, the child labeling a picture of the sun as “night”), which constitutes a serious violation of social norms in Yucatec Maya settings (more than in US settings, [17]). Similarly, waiting several minutes to consume a highly valued food has been described as a more common expectation of young children in Japanese settings than in US settings, making the Marshmallow Test in some sense “easier” for Japanese children than for US children because it is more similar to habits they have built up in the course of their daily lives [16]. In both of these examples—and, presumably, for any task employed to assess an underlying capacity for executive function—children might “underperform” when the task requires them to go against social-cultural norms, and “overperform” when the task aligns well with social-cultural norms. Many of the tasks widely used in developmental psychology were developed in the US and other Western settings by researchers with expertise in these local cultures; we would argue that this is why they are well-calibrated to prevent such concerns about under- or over-performance among US children. When researchers employ these tasks in other settings without adapting them to the local cultural setting, concerns about the local and global validity and reliability of such measurements arise.

Taking this into consideration, the DBN has consistently prioritized developing novel exploratory methods as an attempt to be inclusive of the lived experiences of children across the network. The tasks developed for Wave 1 of this study reflect our first attempt to design tasks that were equally well-suited for children across the many cultural-religious settings represented in the network [1]. As research teams collected Wave 1 data, they were asked to attend closely to the experiences of children in the study and to solicit feedback from local research team members about how it felt to administer these methods and how well children understood what was being asked of them. In preparing to launch a second wave of data collection, the consensus among network members was to prioritize refinements and improvements to the methods over replication of the exact methods used in Wave 1.

An additional consideration was the stress on resources for both the research teams and the participating families. It is well known that longitudinal and cross-cultural research employing interview methods requires ample resources, both within a given wave of data collection and to sustain participation and minimize attrition across data collection time points. The updates and revisions between Waves 1 and 2 of data collection were especially attuned to the amount of time it took for any individual participant and their caregiver to complete the protocol, as well as the amount of time between the two waves of data collection. When providing feedback on Wave 1, research teams frequently reported that the Wave 1 protocol was too long for their sites, often lasting well over an hour and requiring more than one session. Thus, a key priority for Wave 2 was to reduce the length of the protocol to be sensitive to the time investment of our participants and research staff.

We were also restricted in the amount of time we had as researchers to launch Wave 2 data collection due to our grant timeline. Our grant timeline proposed that participants would complete three waves of data collection with Wave 2 and Wave 3 occuring one and two years after their first participation, but the actual amount of time it took to collect Wave 1 data varied across research teams: Some research teams were still collecting Wave 1 data when other research teams needed to begin Wave 2 data collection, because of grant timelines, restrictions on travel schedules, and the availability of research staff. Therefore, this left the network with a limited amount of time (only a few months) to modify and design the Wave 2 protocol, in contrast to the 18 months that were used developing the Wave 1 protocol.

The goal of the current manuscript is to explain our process of modifying the Wave 2 protocol, allowing for greater cultural sensitivity and decrease time investment for researchers and participants, in the hopes that future researchers can gain insights from our experience. Developing this protocol involved balancing the tension between faithful reproduction and responsive modification: In some cases we prioritized maintaining the integrity of the Wave 1 protocol for longitudinal analysis, in others we prioritized refining our methods or asking new questions to be more developmentally or culturally appropriate, and in still others cases, we prioritized decreasing the length of the protocol to relieve the resources required for participation. In all cases, we also recognized the reality of moving quickly to proceed with completing multiple waves of data collection within our grant timeline.

## Materials and methods

The current protocol is designed to address the research questions described above. Research teams were encouraged to conduct Wave 2 in all of the field sites and with all of the cultural-religious samples included in Wave 1 [see 1]; children in the six additional samples added to the network since Wave 1 are completing the Wave 2 Child Protocol described in the current manuscript. As a network, we made this decision because we felt the Wave 2 Child Protocol more appropriately balanced the tensions highlighted above. Nevertheless, because this was the first wave of data collection in these settings and we were interested in including the most comprehensive set of demographic characteristics, we invited caregivers to complete the Wave 1 Parent Survey and the Caregiver Child Conversation Task described in [1].

At the time of submission of the current manuscript, Wave 2 data collection is underway in 41 cultural-religious settings, spanning 16 countries and 12 written languages. These sites are grouped by “intermediary region” according to the United Nations Statistics Divison’s M49 standard geoscheme [18] below.

In North America, the study is being administered in English (and in Spanish as needed) to participants who self-identify as Protestant, Catholic, members of the Church of Jesus Christ of Latter-day Saints, Jewish, Muslim, or religiously unaffiliated living anywhere in the United States (PIs: Rebekah Richert and Kathleen Corriveau).

In Latin America and the Caribbean, the study is being administered in Spanish to Catholics and Evangelical Protestants living in the Mantaro Valley, Peru (PI: Katherine McAuliffe).

In Europe, the study is being administered in English to Catholics and Protestants living in Northern Ireland (PI: Jocelyn Dautel; co-PIs: Laura K. Taylor, Aidan Feeney, and John Coley); in English to Catholics and Protestants living in the Republic of Ireland (PI: Jocelyn Dautel; co-PIs: Laura K. Taylor, Aidan Feeney, and John Coley); in English to Christians, Muslims, and religiously unaffiliated families living in England (PI: Emily Burdett; co-PIs: Helen Elizabeth Davis and Michael Gurven); and in Greek to Greek Orthodox Christians living in and around Athens, Patras, and Tinos (Cyclades) Greece (PI: Irini Skopeliti; co-PIs: Ageliki Nicolopoulou and Natassa Kyriakopoulou).

In sub-Saharan Africa, the study is being administered in Rutooro to Anglicans and Catholics living in and around Kabarole, Uganda (PI: Katherine McAuliffe); and in Xitsonga and Sepedi to Christians with a range of syncretic traditional Southern African beliefs and practices living in the rural villages surrounding Tzaneen, South Africa (PI: Amanda Tarullo; co-PIs: Denise Evans and Peter Rockers).

In Western Asia, the study is being administered in Arabic (with limited code-switching to French and English as needed) to members of the Druze faith living in and around the Chouf Mountains (Lebanon), and to Maronite Catholics, Orthodox Christians, Shia Muslims, and Sunni Muslims living throughout Lebanon (PI: Tamer Amin; co-PI: Maliki E. Ghossainy).

In Southern Asia, the study is being administered in Hindi to Hindus and Muslims living in Vadodara, Gujarat, India (PI: Mahesh Srinivasan; co-PIs: Audun Dahl and Gil Diesendruck).

In South-eastern Asia, the study is being administered in English to Buddhists and Muslims living in Singapore (PI: Tamar Kushnir; co-PIs: Yue Yu, Xin [Alice] Zhao, and Shaun Nichols); in Indonesian to Muslims, Christians (Protestants and Catholics), and Buddhists living in Jakarta, Indonesia, and to Hindus living in Bali, Indonesia (PI: Florencia Anggoro; co-PI: Benjamin Jee); in Malay to Christians living in Tenom, East Malaysia, and in Mandarin Chinese to Buddhists in Kuala Lumpur, Malaysia (PI: Frankie Fong; co-PI: Mark Nielsen).

In Eastern Asia, the study is being administered in Mandarin Chinese (written in Simplified Chinese) to religiously unaffiliated people living in and around Shanghai, Mainland China (PI: Tamar Kushnir; co-PIs: Xin [Alice] Zhao, Yue Yu, and Shaun Nichols); and in Mandarin Chinese (written in Traditional Chinese) to Buddhist families and Taoist/Yiguandao families living in and around Hsinchu, Taiwan R.O.C. (PI: Kathleen Corriveau; co-PI: Eva Chen).

Finally, in Oceania, the study is being administered in English to Christians and non-religious families in Brisbane, Australia (PI: Frankie Fong; co-PI: Mark Nielsen).

All research teams aim to collect data from as many Wave 1 participants as possible for each cultural-religious sample, with the understanding that attrition in multi-time point data is unavoidable. As in the first wave of data collection, all other recruitment and data collection strategies vary across samples; these decisions are made by field site leaders in close consultation with the core leadership team [see 1].

Our study protocol for the first wave of data collection provided details of the implementation of that study in the United States [1]; the current manuscript provides a detailed description of the adaptation and implementation of this protocol in Lebanon for the five religious groups included in Wave 2 data collection: members of the Druze faith, Maronite Christians, Orthodox Christians, Shia Muslims, and Sunni Muslims. In this manuscript we focus on Lebanon as the example field site because it is one of the largest and most varied field sites in the DBN, including contrasts both between religions (e.g., Christianity versus Islam) and within the same religion (e.g., Sunni versus Shia Islam, Maronite Catholicism versus Orthodox Christianity). Importantly, by featuring Lebanon, we provide five examples of how the protocol was implemented outside of “WEIRD” [12] and “Minority World” [13] settings; this reflects the aspiration of researchers in the network to decenter the United States and Western Europe as the base for cultural comparison and as the “defaults” for understanding child development [19,20].

The following section (“Samples”) was drafted by the leaders of the research team in Lebanon: Tamer Amin (PI), Maliki E. Ghossainy (co-PI) and Ghadir Soueidan (Lead Researcher).

### Samples

#### Sample size (Lebanon).

In Wave 1, we set a target sample of 40 children and their parents/caregivers for each of the six religious groups recruited for Wave 1 in Lebanon. This was the largest sample we could imagine attaining given the Lebanon data collection staff and budget and local circumstances at the time of Wave 1 data collection. All families who participated in Wave 1 and indicated willingness to be contacted again for further participation are invited for Wave 2 participation 9–15 months after the initial Wave 1 child interview was conducted, with one exception: Due to challenges and delays in recruitment of Protestant Christian families in Wave 1, we are not including this small religious minority group in our Wave 2 sample. This yields a total possible sample of 200 children (across five religious samples) for Wave 2 data collection in Lebanon. Recruitment for this sample began on May 1, 2023.

#### Eligibility, inclusion, and exclusion criteria (Lebanon).

Lebanon samples are recruited through the American University of Beirut in Beirut, Lebanon. To be eligible for this multi-time point study, a family must live in Lebanon, one of the parents must hold Lebanese nationality, and the child participating in the study must have spent at least three quarters of their life in Lebanon. At the time of their participation in Wave 1, children were required to be between the ages of 4 years 0 months and 10 years 11.99 months. Children must be native speakers of Lebanese Arabic (the language in which the interview is conducted), and parents/caregivers must be able to read and respond to the Parent/Caregiver Survey in writing in Modern Standard Arabic. Participating parents/caregivers must be parents or legal guardians of the participating child; multiple siblings from the same family are allowed to participate. To be included in Wave 2, at Wave 1 parents/caregivers must have reported that children were being raised in one of the following religious traditions: the Druze faith, Maronite Christianity, Orthodox Christianity, Shia Islam, and Sunni Islam.

Prior to participating in Wave 1, parents/caregivers interested in participating in the study were asked to indicate the family’s religious affiliation. This determined which version of the Child Protocol and Parent/Caregiver Survey was administered to the family at Wave 1; Wave 2 is matched to this Wave 1 decision. In cases where the parents/caregivers are affiliated with different religious groups, the parent/caregiver was asked in this pre-Wave 1 questionnaire to indicate the religious affiliation the child identifies with or is most familiar with. At Wave 2 (as in Wave 1), the child is given a version of the protocol that features stimuli drawn from the religion they are most familiar with; in cases of mixed-religion households, the parent/caregiver whose religious affiliation best matches this selection is asked to complete the Parent/Caregiver Survey.

Although the families recruited for Wave 1 varied in their engagement with religious practices, only those families who explicitly identified as members of a particular religious group were recruited for the study.

The research team working in Lebanon has adopted the following exclusion criteria for all waves of data collection. First, children who do not appear to understand the majority of the Child Protocol are excluded, as are children who lose interest or are reluctant to engage productively, and ultimately prefer to opt out after initially providing consent to participate. Second, children whose parents/caregivers either report a formal diagnosis of a developmental disorder or report their own concerns about their child’s comprehension, verbal expression, working memory, or other factors are also excluded from the sample. Third, children whose sessions are frequently interrupted (e.g., by family members making suggestions about how the child should answer) are excluded from the sample; judgments of the frequency and severity of these interruptions are made by data collectors. We have no current plans to exclude data at the trial or task level; that is, if a child’s data is included in the dataset our default will be to include all of their data. However, there might be some exceptions to this made on a case-by-case basis during regular meetings of the data collection team. In keeping with the practices employed by the DBN, all decisions regarding exclusion are made by the local research team in Lebanon.

#### Recruitment, data collection, and compensation (Lebanon).

For Wave 1 of this study, participants were recruited through schools, youth groups, and community members who agreed to circulate an invitation to participate in the study to parents and through snow-ball sampling from those already recruited. Recruitment was carried out in multiple regions in Lebanon so as to achieve a diverse sample across the different religious groups. This included Beirut, the Chouf Mountains, the South (the city of Sidon), the North (Keserwan and Akkar), and the Bekaa Valley.

Families who participated in Wave 1 and indicated a willingness to be re-contacted for future waves of data collection are invited to participate in Wave 2 using the contact information that they provided when they initially participated.

All child interviews are conducted in person, usually in the family’s home but sometimes in private spaces of a house of worship or community center or at the American University of Beirut. If a parent/caregiver and the child consent, the interview is video or audio recorded for later analysis. During the interview, the researcher also logs most of the child’s responses in real-time via Qualtrics survey software. After the interview, the designated parent/caregiver is sent a link to the Parent/Caregiver Survey which they complete online via Qualtrics survey software.

To thank participants for their time and their contributions to this study, families are given 10 USD for each participating child upon completion of the Child Protocol. For families with more than one child participating, parents/caregivers receive an additional 10 USD for each child.

### Overview

The protocol consists of two components: the Child Protocol, a set of modified or repeated behavioral tasks for children between the ages of 5–13 years; and the Parent/Caregiver Survey, a brief survey to be completed by children’s parents or other primary caregivers. For the full text of all materials (as adapted for the five target religions included in samples in Lebanon) and Qualtrics survey (.qsf) files, please visit the following OSF repository: https://osf.io/p326e/.

**Fig 1:**
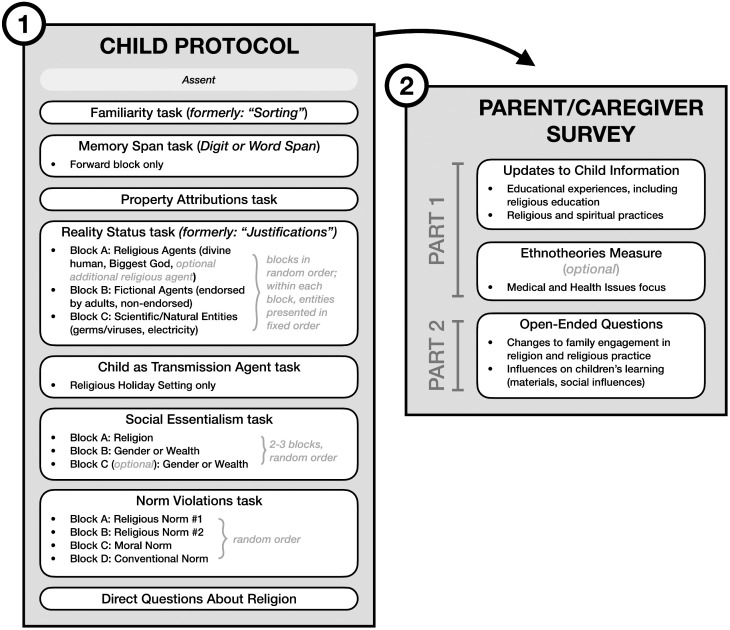
Overview of the full Developing Belief Network Wave 2 study protocol for children (ages 5–13 years) and their parents or other primary caregivers. Within each task in the Child Protocol, blocks are presented in a random order for each child. With respect to the Parent/Caregiver Survey, research teams may opt to administer Part 2 separately from Part 1 if desired (e.g., administering Part 1 via online survey and Part 2 via oral interview).

A primary goal of this multi-time point research project is to document children’s understanding of the various religious entities, practices, and norms they encounter in their own lived experiences. As in the first wave of data collection [described in 1], for each cultural-religious sample we thus tailor our materials to include culturally appropriate stimuli that are likely to be salient to participants in a particular cultural-religious sample, while maintaining some degree of standardization across all samples in the network. Many of these sample-specific adaptations from Wave 1 are carried over into the Wave 2 protocol described in the current manuscript. Here, we present the standard versions of each element of the study protocol; see Figure 1. For each protocol element, we also detail the sample-specific adaptations for the five target religions included in samples from Lebanon, where sample-specific adaptation is complete and data collection is underway.

A general feature of this adaptation relates to the multilingual nature of Lebanese society. As indicated above, the Child Protocol is conducted in Lebanese Arabic and thus only Lebanese children who are proficient in Lebanese Arabic are recruited to participate in the study. However, in Lebanon all children learn a foreign language (either English or French) at school, alongside Arabic, and some subjects (science and mathematics) are taught in that foreign language. Moreover, many families are multilingual, with a foreign language (usually English and/or French), often playing a role in some aspects of everyday communication. Therefore, it is not uncommon for young children to use Lebanese Arabic as a primary language, but for some topics to be more commonly discussed in a foreign language. Accordingly, the Lebanon team accommodates such language “switching” by including both English and French translations for some key terms/phrases to provide the child with an alternative route to comprehend the intended concept if the Lebanese Arabic word appears unfamiliar (e.g., “germs” or “les germes” are offered alongside the Arabic word “جراثيم”). Note that no explanations of the unfamiliar Arabic words are provided to any child.

### Child protocol

#### Familiarity task (formerly “Sorting task”).

***Rationale for retention and revision of task:*** As in Wave 1, the Wave 2 Child Protocol begins with a Familiarity task (referred to as the “Sorting task” in [1]), designed to assess children’s familiarity with religious agents, fictional agents, and natural entities.

Including a familiarity assessment in all waves of data collection allows for observation of age-related changes in the recognition of targeted religious concepts at the individual level, complementing the cross-sectional insights afforded by the Wave 1 dataset alone. In Wave 2, the Familiarity task also serves as a screening tool, both within a testing session (allowing us to skip over extended questions about agents and entities with which an individual child is unfamiliar) and for future analyses (allowing us to explore variability between children who did versus did not indicate familiarity with a given agent/entity).

The Wave 2 Familiarity task includes the agents and entities that are featured in later tasks in the Wave 2 protocol, rather than the full range of items included in the Wave 1 Sorting task. This allows us to track changes in familiarity within subjects for agents of particular interest to researchers, while reducing the overall length of the protocol and eliminating overlapping questions between the Familiarity task and the Reality Status task (see “Reality Status task,” below).

***Task design and procedure:*** To begin the task, the researcher says, “We’re going to talk about a bunch of different things. Your first job is to tell me if you’ve ever heard of the thing I mention.” Next, the child is presented with two practice trials designed to familiarize children with the task format and to establish that the task can include both familiar and unfamiliar agents. All examples from the child protocol are in Lebanese Arabic, the language in which the Child Protocol was conducted; as a result, the words written in Arabic may differ from Modern Standard Arabic. One practice trial involves a familiar agent (e.g., “ الحكما/الدكاترة,” “doctors” in Lebanese Arabic), and the second practice trial involves the use of a nonsense word that children should indicate is unfamiliar (e.g., “ الهع خع,” “el-ha3kha3”, a nonsense word in Lebanese Arabic). These practice trials are presented in a random order. Finally, the child is presented with six to seven test trials in a random order. On each trial, the child is asked “Have you ever heard of [target agent/entity]?” (response options: “yes” or “no”).

To preserve cultural and religious specificity and appropriateness while maintaining some degree of standardization across samples, each version of the protocol includes a total of six to seven target agents/entities within three categories, as follows.

Each version of the protocol contains two to three religious agents that are widely considered “real” in the cultural-religious setting, matched to the religious agents included in the Wave 1 protocol. This includes one prominent human or human-like religious figure, such as a divine human or a human prophet (e.g., Jesus, the Prophet Muhammad), referred to in this manuscript as Religious Agent 1 (and matched to Agent 1 in the Wave 1 protocol for that sample). It also includes the “Biggest” god in that cultural-religious setting [e.g., God, Allah; see 21], referred to in this manuscript as Religious Agent 2 (again, matched to Agent 2 in that sample’s Wave 1 protocol). Teams can also opt to include an additional prominent religious being in this task, referred to in this manuscript as Religious Agent 3. These agents are also featured later in the protocol in the Property Attributions and Reality Status tasks (described in later sections).

Each version of the protocol also includes two fictional agents widely considered “fictional” or “fantastical” in the cultural-religious setting. These fictional agents are a subset of the fictional agents included in Wave 1. Research teams were advised to choose one agent that is culturally endorsed for children by adults—that is, when adults talk about the agent around children, they tend to use language consistent with it being real (e.g., “بابا نويل/سانتا كلوز” [“Papa Noel/Santa Claus”]); and one agent that is not typically endorsed even for children—that is, an agent for which adults do not go to any special efforts to “make real” for children (e.g., “الغول” [“Ghoul/Monster”]).

Finally, each version of the protocol includes two target entities that are intended to be more natural or scientific in nature. One of these target entities is germs, viruses, or culturally appropriate equivalents (in Lebanon: “الجراثيم;” the research team also provides French or English translations to children as needed); this is matched to the “scientific entity unobservable by the naked eye” featured in Wave 1. The second target entity—newly introduced in Wave 2—is electricity (in Lebanon: “الكهربا”), selected because it is also considered unobservable by the naked eye in previous research [22,23]; the consensus among research team leaders was that this is a term that children would be familiar with in most if not all field sites.

***Adaptation for samples in Lebanon:*** The final set of agents featured in the Wave 2 Familiarity task for the five samples from Lebanon is presented in Table 1.

**Table 1:** Sample-specific item selection for the Familiarity task, for the five samples from Lebanon. See main text for descriptions of network-wide guidelines for item selection.

	Religion				
	*Druze faith*	*Maronite Christianity*	*Orthodox Christianity*	*Shia Islam*	*Sunni Islam*
***Religious agents***					
1	Nabi Ayoub (the Prophet Job)	Jesus	Jesus	the Prophet Muhammad	the Prophet Muhammad
Arabic	النبي أيوب	يسوع المسيح	يسوع المسيح	النبي محمد	النبي محمد
French	–	–	–	–	–
English	–	–	–	–	–
2	Allah	Allah	Allah	Allah	Allah
Arabic	الله	الله	الله	الله	الله
French	–	–	–	–	–
English	–	–	–	–	–
3	Sheikh El Akl	Virgin Mary	Virgin Mary	Imam Ali	Imam Ali
Arabic	شيخ العقل	مريم العذرا	مريم العذرا	الإمام علي	الإمام علي
French	–	–	–	–	–
English	–	–	–	–	–
***Fictional agents***					
1	Santa Claus	Santa Claus	Santa Claus	Santa Claus	Santa Claus
Arabic	بابا نويل	بابا نويل	بابا نويل	بابا نويل	بابا نويل
French	Papa Noel	Papa Noel	Papa Noel	Papa Noel	Papa Noel
English	Santa Claus	Santa Claus	Santa Claus	Santa Claus	Santa Claus
2	monster	monster	monster	monster	monster
Arabic	الغول	الغول	الغول	الغول	الغول
French	monstre	monstre	monstre	monstre	monstre
English	monster	monster	monster	monster	monster
***Natural Entities***					
1	germs	germs	germs	germs	germs
Arabic	الجراثيم	الجراثيم	الجراثيم	الجراثيم	لجراثيمl
French	germes	germes	germes	germes	germes
English	germs	germs	germs	germs	germs
2	electricity	electricity	electricity	electricity	electricity
Arabic	الكهربا	الكهربا	الكهربا	الكهربا	الكهربا
French	–	–	–	–	–
English	–	–	–	–	–

Notes: For each item, the numbered row provides an English translation of the item presented to children. Below that we provide the item in Arabic, as presented to children, as well as French and English versions of the item if they were also available to children to provide an alternative route to the intended concept if the Lebanese Arabic word seemed unfamiliar. (See main text, “Overview.”). If cells are blank (“-”), this indicates that there was no English or French version of the item available to children.

***Planned analyses:*** This task primarily serves as a familiarity check for other tasks in the protocol (see Reality Status task, below).

Nonetheless, as in Wave 1, we also hypothesize that children’s familiarity with the target agents/entities included in this task will increase with age, and that this increase will be more pronounced for religious agents. Beyond this, we plan to assess age-related changes in familiarity via within-subjects models of changes in familiarity judgments across waves (for target entities that were included in more than one wave of data collection).

#### Memory span tasks.

***Rationale for retention and revision of task:*** As in Wave 1, the Wave 2 Child Protocol also includes a working memory span task. Memory span tasks are widely used as a measure of short term auditory memory, one aspect of broader cognitive development and executive function [24,25].

Wave 1 featured both “digit span” and “word span” adaptations of the “Memory for Digit Span assessment: Digits Forward and Digits Backward” components of the Wechsler Intelligence Scales for Children-Revised (WISC-R) [26]. To reduce the overall length of the Wave 2 protocol we include only the “forward” set of trials, for only one of the two versions of the task (digit span or word span), depending on which version was more culturally appropriate.

Although the length of the protocol restricts the ability to incorporate a full battery of measures of general cognitive development or executive function, we include an assessment of working memory at each timepoint to allow researchers to better understand how changes in children’s responses across waves of data collection might be shaped by domain-general changes in working memory (known to be associated with other general cognitive developmental changes) versus domain-specific changes in religious beliefs and behaviors (due, e.g., to religious experiences or education that the child may have received between timepoints).

***Task design and procedure:*** The Wave 2 Child Protocol includes the “forward” block of one version of the Memory Span task, either the “digit span” version, featuring the numbers 1–9, spoken in the language of study administration; or the “word span” version, featuring nine familiar words roughly matched in syllable length [see 1]. In Wave 2, research teams were given the option to select either digits or words; to date, all research teams have decided to use the digit span.

Our administration of this forward digit span task is identical to the description in [1]. We describe it briefly here for completeness; see the previous manuscript for more details and discussion.

At the beginning of the Memory Span task, the researcher provides an introduction and set of practice trials, specially designed for use in the range of samples represented by the DBN. The researcher then begins the test trials, following the instructions for the “Memory for Digit Span assessment: Digits Forward” component of the WISC-R [26]. There are a total of 8 “levels” test trials a child can complete, fixed in ascending order of difficulty (Level 1 = sequences of two digits; Level 8 = sequences of nine digits). The researcher reads each sequence aloud at a pace of approximately 1 digit per second; then the child attempts to repeat back the sequence in the same order. Children are not given any feedback about their responses, regardless of accuracy. Responses that are incomplete and responses in which children begin the sequence incorrectly but then correct themselves are both counted as incorrect. If the child repeats at least one of the two sequences back correctly on a given level, they move onto the next level of the task. The task ends when the child provides two incorrect responses within the same level.

***Adaptation for samples in Lebanon:*** This task does not require any sample-specific customization, beyond translation.

***Planned analyses:*** Analyses of the Memory Span task will follow the standards for analyzing “Memory for Digit Span assessment: Digits Forward” components of the WISC-R [26], including analyses of assessing individual differences and developmental differences.

We anticipate that researchers will apply both single-timepoint (cross-sectional) and multi-time point analysis techniques to the data from this task. As in Wave 1, analyses might include examinations of relationships between responses to this task and responses to other tasks included in the Wave 2 Child Protocol within a given cultural-religious sample; assessments of the validity of the memory span task in a given field site; and careful explorations of how responses to the memory span task might shed light on any observed similarities and differences within and across samples for other tasks in the Child Protocol. Applying this logic across timepoints, analyses might include examinations of the degree to which individual-level changes in memory span performance across timepoints might explain changes in responses to other parts of our protocols.

#### Property Attributions task

***Rationale for retention and revision of task:*** As in Wave 1, the Wave 2 Child Protocol includes a Property Attributions task, designed to assess children’s concepts of religious agents, with a particular focus on how these agents are understood to be constrained or unconstrained by the “laws” of folk physics, folk biology, folk psychology (including both cognitive-epistemic and social-emotional aspects of folk psychology), and folk sociology. For similar tasks, see [27–29].

Including such an assessment in all waves of data collection allows us to observe age-related changes in concepts of religious agents at the individual level, to complement the cross-sectional insights afforded by the Wave 1 dataset alone. For this task in particular, we consider within-subjects studies of development to provide important insights into the ways in which children’s understanding of the physical, biological, psychological, and sociological constraints on religious beings might depend on children’s personal exposure to religious doctrine and representational practices (e.g., hearing stories about Jesus walking on water; being told that Allah is completely different from humans; seeing pictures and statues), as well as their personal engagement in religious practices (e.g., opening the door for Elijah during Passover, leaving offerings of food for ancestors).

Researchers across many of the field sites and samples included in the network report that the Wave 1 Property Attributions task was very long for children, with many participants losing interest or focus over the course of the task. To address this in Wave 2, we adopt a “planned missingness” approach [30]: For each child, we randomly select two of the four items included within each domain, reducing the length of the task by 50% for each child. This approach allows us to gather data on all domains for each of the target agents at each time point from each child, while distributing the individual properties within domains assessed at Wave 2 across the whole sample.

***Task design and procedure:*** The Wave 1 Property Attributions task consisted of a 20-item close-ended battery intended to assess participants’ representations of the physical, biological, cognitive-epistemic, social-emotional, and sociological properties and constraints of two to three religious agents, as compared to the properties and constraints of ordinary human agents. For a complete description, including the full set of items, see [1].

In Wave 2 each child is shown a random subset of two of the four items for the physical domain, two of the four items for the biological domain, two of the four items for the cognitive-epistemic domain, two of the four items for the social-emotional domain, and two of the four items for the sociological domain. Put another way, each child assesses half of the 20 original items, and each of the 20 original items are assessed by roughly half of the children within each sample.

For each item, the child responds to a prompt about an ordinary human (“a person”), and two to three religious agents (Religious Agent 1, Religious Agent 2, and, if relevant, Religious Agent 3; see description under “Familiarity task” above). These religious agents are matched to the agents featured in Wave 1; they are also featured in the Wave 2 Familiarity task (described in a previous section) and in the Wave 2 Reality Status task (described in a later section). If a child has indicated in the Familiarity task that they are not familiar with one of these agents, this agent is omitted from the Property Attributions task for that child.

***Adaptation for samples in Lebanon:*** The set of religious agents featured in the Property Attributions task for the five samples from Lebanon is described in the “Familiarity task” section above (see Table 1, Religious Agents).

***Planned analyses:*** For single-timepoint (cross-sectional) analyses, see the planned analyses in [1]. We note, however, that in Wave 2 each child contributes only half of the data points that children contributed in Wave 1, per the “planned missingness” approach described above. This makes item-level analyses even more critical for interpreting response patterns and age-related differences.

Bearing in mind the constraints just described, multi-time point analyses might be conducted either at the item level, with each participant contributing data for a subset of items in the original Wave 1 protocol; or at the level of the domain (physical, biological, and so on), with each participant contributing a “score” for each domain.

Pooling across the diverse religious traditions represented in the network, we hypothesize that, on average, attributions of human-like properties to religious agents will decrease across waves; that is, as children grow older they will be more likely to say that religious agents will violate constraints. To test this hypothesis, we will examine the main effect of participant age within subjects, considering only religious agents. We also anticipate that, on average, children’s perceptions of the *difference* between religious agents and the human will increase across waves; to test this, we will also examine a statistical interaction between agent type (religious versus human) and participant age within subjects.

However, as the examples from Lebanon illustrate, we expect substantial variability across religious groups and religious agents—not only in the degree of these (hypothesized) developmental trends, but also in their direction. For example, in many Muslim settings, the Prophet Muhammad is not considered “divine” from a theological perspective, so we would expect increased recognition of his human status with the acquisition of more religious knowledge. In this case, we would predict that as Muslim children grow older they will be *less* likely to say that this particular religious agent (the Prophet Muhammad) will violate constraints, and that the perceived difference between the Prophet Muhammad and the (ordinary) human agent will *decrease* across waves. Exploring similarities and differences in these patterns across cultural-religious settings will be critical to making sense of, adding nuance to, and noting exceptions to any general trends in the pooled sample.

Additional exploratory analyses will investigate whether these hypothesized trends vary across domains (e.g., whether these age-related changes are more pronounced in the physical and biological domains than the cognitive-epistemic, social-emotional, and sociological domains) and across agents (e.g., whether these age-related changes are more pronounced for “Big Gods” than for other religious/supernatural agents).

#### Reality Status Task (formerly “Justifications task”).

***Rationale for retention and revision of task:*** As in Wave 1, the Wave 2 Child Protocol includes a Reality Status task (referred to as the “Justifications task” in [1]). This task is designed to evaluate children’s judgments about the reality status of a variety of religious, fantastical, and natural entities, as well as their understanding of their own source knowledge regarding these entities (i.e., how they might justify their reality judgments). For similar tasks, see [23,27–29,31–33].

Including the Reality Status task in all waves of data collection allows us to observe age-related changes in concepts of religious agents at the individual level, to complement the cross-sectional insights afforded by the Wave 1 dataset. Again, we consider children’s concepts of religious agents to be particularly sensitive to personal experience, personal exposure to testimony and religious doctrine, and personal engagement in religious practices—all of which underline the importance of within-subjects work across multiple time points.

We have made several changes to the task to address feedback raised by network members. First, to address questions of how children’s reasoning about religious agents compares to their reasoning about other agents and entities, we now ask these questions not only about religious agents, but also about the fictional agents and natural entities in the Wave 2 Familiarity Task. To compensate for the additional time this added to the task, we exclude the block of questions included in Wave 1 focusing on children’s reasoning about “normal, everyday people.” Second, we focus primarily on forced-choice responses, rather than open-ended responses; this is intended to address concerns raised by network members about systematic variability across field sites in children’s willingness and comfort providing open-ended responses to these kinds of questions. Finally, this task includes one new question (and a follow-up) designed to assess an additional aspect of children’s learning about these entities: their strategy for seeking further information about the entities in question.

***Task design and procedure:*** In Wave 2, the Reality Status task features all of the target agents/entities included in the Familiarity Task (described in an earlier section): two to three religious agents (Religious Agent 1, Religious Agent 2, and, if relevant, Religious Agent 3; see description under “Familiarity task” above); two fictional agents, one of which is generally endorsed by adults when they speak to children, while the other is not; and two target entities that are intended to be more natural or scientific in nature (“germs” or “viruses”, and “electricity”). If a child has indicated in the Familiarity task that they are not familiar with one of these agents/entities, it is omitted from the Reality Status task for that child.

The task consists of a separate block of questions for each agent/entity. The two to three religious agents are always presented one after another (Religious Agent 1, followed by Religious Agent 2, followed by Religious Agent 3 if applicable in the sample); the two fictional agents are always presented one after another (endorsed, followed by non-endorsed); and the two natural entities are always presented one after another (germs/viruses, followed by electricity). Beyond this, the order of presentation is random: Some children see the blocks about religious agents first, others see the blocks aboutfictional agents first, and so on. Each block begins with a very brief introduction—“Now I’m going to ask you some questions about [agent/entity]”—followed by questions about children’s judgments about the reality status of the agent. First, the researcher asks the child to make a *reality judgment*: “Is/are [agent/entity] real or not real?” Then the researcher asks the child to assess their *certainty* about this reality judgment by asking, “How sure are you that [agent/entity] is/are [child’s answer: real/not real]? A little bit sure, or really sure?” This is followed by an assessment of the child’s *source knowledge* regarding this reality judgment: “How do you know that [agent/entity] is/are [child’s answer: real/not real]?” The final question in this set of questions is about the child’s sense of *community consensus* regarding this reality judgment: “Would most people in your [city/town/village] agree with you and say that [agent/entity] is/are [child’s answer: real/not real]?” This set of questions is identical to the questions used in Wave 1.

Finally, the child is asked two additional questions designed to shed light on their information seeking regarding the target agent/entity: “If you wanted to know more about [agent/entity], what would you do? Would you read, ask someone, or figure things out for yourself?” This question is followed by an open-ended question designed to prompt children to elaborate on their previous response; as appropriate, the experimenter asks one of the following questions: “What would you read?” or “Who would you ask?” or “How would you figure things out?”

***Adaptation for samples in Lebanon:*** The set of religious agents, fantastical entities, and natural entities featured in the Reality Status tasks for the five samples from Lebanon is described in the “Familiarity task” section above (see Table 1).

***Planned analyses:*** For single-timepoint (cross-sectional) analyses, see the planned analyses in [1,2]. We anticipate implementing similar coding schemes to characterize trends and variability in the content of children’s responses (e.g., identifying certain content as “religious”; counting mentions of “God” or other religious agents; coding different types of explanations) and their manners of speaking (e.g., noting the use of generic sentences). For similar approaches, see [32,34].

Wave 2 offers a new opportunity to compare children’s responses to religious agents versus fantastical agents versus natural entities; other single-timepoint analyses will focus on these differences.

Additional single-timepoint analyses will focus on children’s forced-choice responses to the new set of questions of the form, “If you wanted to know more about [target agent/entity], what would you do? Would you read, ask someone, or figure things out for yourself?” Such analyses will be exploratory in nature.

Multi-time point analyses will only be possible for the target agents/entities included at both time points: Religious Agent 1, Religious Agent 2, and Religious Agent 3 (where applicable). We hypothesize that, for all open-ended questions, children will offer richer and more detailed examples, descriptions, and explanations of what they know and how they know it at Wave 2 [see 32, 35, 36]. To test this hypothesis, we will examine the main effects of participant age within subjects using any relevant coding schemes we have developed to quantify and characterize children’s responses.

#### Child as Transmission Agent task.

***Rationale for retention and revision of task:*** The Wave 2 Child as Transmission Agent task is a modified version of the qualitative, exploratory task used in [1], designed to capture the ways in which children are themselves vectors of religious information transmission. Wave 1 included 2 separate blocks related to the celebration of a religious holiday versus practices at home; in Wave 2, we include only the religious holiday block of this task.

As research teams began collecting data for Wave 1, they reported a wide variety of responses to the open-ended questions included in this task in Wave 1. In some settings, children had less experience engaging in one-on-one conversations with adults, especially conversations involving open-ended and abstract questions, and were hesitant to offer more than a few words in response to our questions. In other settings, such conversations were well-received and highly familiar to children. In response to this feedback, in Wave 2 we eliminate one open-ended question and employ a forced-choice format for another question, yielding a slightly shorter task that is better suited to the range of cultural-religious settings represented by the DBN, while allowing for within-subjects exploration of changes in how children talk about their religious holidays over time.

***Task design and procedure:*** As in Wave 1, children are introduced to the task with the following script: “Let’s imagine [character] is a new kid in your neighborhood who is younger than you who doesn’t know what happens at your house. Now let’s say it’s a special day like [religious holiday]. [Character] needs your help to remember what you do when celebrating this special holiday.”

Children are then invited to provide open-ended instructions and explanations about the things that the character should do to observe the religious holiday the right way and prompted to explain why the character should behave this way. For each cultural-religious sample, Wave 2 features the same sample-specific holiday and the same two sample-specific prompts about that holiday that were featured in Wave 1; see Table 2 for examples from samples in Lebanon.

**Table 2:** Sample-specific item selection for the Child as Transmission Agent task, for the five samples from Lebanon.

	Religion				
	*Druze faith*	*Maronite Christianity*	*Orthodox Christianity*	*Shia Islam*	*Sunni Islam*
Character	Sara	Sara	Sara	Sara	Sara
	سارة	سارة	سارة	سارة	سارة
Target religious group	Druze	Maronite	Orthodox	Shia	Sunni
	درزي/ة	ماروني/ة	أورثوذوكس	شيعي/ة	سني/ة
Holiday	Going to Nabi Ayoub's (the Prophet Job's) shrine	Easter	Easter	Ramadan	Ramadan
	رايحة عمقام النبي أيوب	الفصح	الفصح	رمضان	رمضان
Item #1	What should Sara wear if she goes with you to Nabi Ayoub's (the Prophet Job's) shrine?	Is there anything Sara should or should not do in the weeks before Easter?	Is there anything Sara should or should not do in the weeks before Easter?	What should Sara do if she’s invited for Iftar?	What should Sara do if she’s invited for Iftar?
	شو لازم تلبس سارة إذا رايحة معكن عمقام النبي أيوب؟	في شي سارة لازم تعملو أو مش لازم تعملو بالأسابيع يلي قبل عيد الفصح؟	في شي سارة لازم تعملو أو مش لازم تعملو بالأسابيع يلي قبل عيد الفصح؟	شو لازم تعمل سارة إذا معزومة لعندكن عالإفطار؟	شو لازم تعمل سارة إذا معزومة لعندكن عالإفطار؟
Item #2	What should Sara do or say before going into the shrine?	What should Sara do on Easter Sunday?	What should Sara do on Easter Sunday?	What should Sara do or say if she comes over for Eid El-Fitr?	What should Sara do or say if she comes over for Eid El-Fitr?
	شو بتقول أو بتعمل قبل ما تفوت عالمقام؟	شو لازم تعمل سارة بنهار عيد الفصح؟	شو لازم تعمل سارة بنهار عيد الفصح؟	شو لازم تقول أو تعمل سارة إذا إجت لعندكن ععيد الفطر؟	شو لازم تقول أو تعمل سارة إذا إجت لعندكن ععيد الفطر؟

As in Wave 1, children are then asked to advise the character about what to do if she/he wanted to learn more about the holiday in question. Rather than asking this in an open-ended format, in Wave 2 we instead provide three options and ask children to choose among them: “If [character] has more questions about what to do when celebrating [religious holiday], what should she/he do? Should [he/she] read, ask someone, or figure things out for [his/herself]?” Then children are then prompted to expand on their selected method of information-seeking: “What should [he/she] read?”, “Who should [he/she] ask?”, or “How should he/she figure things out?”

Finally, as in Wave 1, children are asked whether the character is a member of the target religious group or not. Depending on the sampling plans in place at a given field site, the target religious group might correspond to the participating child’s own religious group, to a religious group with which the child’s parent has indicated that they are familiar, or to a religious group that is prevalent in the participating child’s local context. (Note that the religious group named in this task is also featured in two other tasks in the protocol: the Norm Violations task and the Social Essentialism task, described in the following sections).

***Adaptation for samples in Lebanon:*** The final set of properties featured in the Child as Transmission Agent task for the five samples from Lebanon is presented in Table 2.

***Planned analyses:*** Coding schemes developed for analysis of Wave 1 will be applied again to the analysis of open-ended questions. These coding schemes have not yet been developed but could include coding schemes related to identifying certain content as “religious”; counting mentions of “God” or other religious agents; coding different types of explanations for why the character should or should not behave in a certain ways, as well as various manners of speaking [e.g., noting the use of generic sentences; see 1].

We hypothesize that, with increased age and more experience with religious holidays in their own lives between waves, children will be more likely to spontaneously mention religious content (e.g., rules, practices, rituals) and offer richer and more detailed responses at Wave 2, relative to Wave 1 [see 37, 38].

#### Social Essentialism task.

***Rationale for retention and revision of task:*** As in Wave 1, the Wave 2 Child Protocol includes a Social Essentialism task, designed to gauge the ways in which children do or do not essentialize social groups based on religion, gender, and wealth [see 39-41].

Our goal in including such an assessment in all waves of data collection is to chart changes in essentializing at the individual level. We consider within-subjects exploration to be particularly important, because we speculate that essentializing may be driven in large part by children’s personal experiences, by events in their family lives, and by their observations of their immediate environments (including the ways in which people talk about these social groups around the child)—all of which would result in large individual differences that might be washed out by an overreliance on cross-sectional samples.

At the same time, the process of collecting Wave 1 data across the many field sites and samples included in the network has prompted substantial changes to this task. The goals of our changes are twofold: First, we aim to improve children’s comprehension of the task and the interpretability of their answers; second, we aim to address the many aspects of social (and biological) essentialism that have been assessed in previous work.

The Wave 2 version of this task omits two of the four questions included in Wave 1: one about physical insides and one about group membership at birth. With respect to the physical insides question (“Is it possible to tell whether a person is [non-reference group] or [reference group] just by looking inside their body, like by looking at their blood and bones?”), children in some samples may have less experience with tools that can be used to see people’s insides (e.g., through blood draws, X-rays) compared to children in other samples, which makes it difficult to pool responses across samples responsibly. With respect to the question about group membership at birth (“Are people born that way? Like, [non-reference group] are born [non-reference group] and [reference group] are born [reference group]?”), children might endorse what we initially intended to be an essentialist response option (that people are “born that way”) without an actual commitment to essentialist logic (e.g., children might think that people who are poor now were “born that way” because of structural factors and not because of any inherent or heritable essence).

We have replaced these two items with two new questions about the homogeneity of the social categories in question [see 42, 43]—an important aspect of social essentialism which is not assessed directly in the Wave 1 version of this task.

In addition, Wave 2 includes follow-up questions to the Wave 1 item about the possibility of transformation (a person changing group membership), to clarify the nature of children’s responses. These are intended to differentiate between essentialist (or “internalist”) explanations, which appeal to internal properties inherent and perhaps essential to membership a category (e.g., that members of Category X are genetically predisposed to excel in math), versus structuralist explanations, which instead focus on stable external constraints that systematically apply to category members (e.g., that members of Category X are systematically given opportunities to develop their math skills) [see 44–47].

The remaining item from Wave 1 about spiritual insides has been retained without modification.

To address the need to reduce the overall length of the protocol as a whole, research teams can opt to include two instead of three blocks of this task (i.e., to assess children’s tendency to essentialize religious groups plus either gender-based or wealth-based groups, rather than all three social categories).

The Lebanon research team extended this task to include a block about language (e.g., assessing the essentialization of French speakers vs. English speakers). This was a sample-specific addition to the procedure and is not considered part of the network-wide standard Child Protocol.

***Task design and procedure:*** The Wave 2 Social Essentialism task consists of two or three blocks, presented in random order, focusing on at least two of the following three social categories: religion, gender, and wealth. All research teams must include the religion block in their adaptation of the protocol; beyond this, research teams may opt to include one or both of the remaining social categories.

As in Wave 1, at the beginning of each block, children are shown silhouettes of two groups of people side by side; the researcher identifies these groups as members of contrasting social categories. One group is considered the “reference group” (generally either the child’s ingroup, a majority group in the local setting, or a group of higher social status in the local setting) and one the “non-reference group” (the child’s outgroup, a minority group, or a group of lower social status).

For the religion block, the reference and non-reference groups vary across samples, and are matched to the groups used for each sample in Wave 1. As in Wave 1, this yields a clear ingroup versus outgroup contrast for participating children in some samples, while in other samples, the contrast is between a majority versus minority religious group, or between a higher- versus lower-status religious group.

As in Wave 1, the reference group in the gender block is the participating child’s own gender (e.g., “girls”), and the non-reference group is the contrasting binary gender (e.g., “boys”). If the participating child does not identify as a boy or a girl, or if the participating child’s gender is unknown, the child is shown the “girl” version of the task.

As in Wave 1, the reference group in the wealth block is always “rich people,” and the non-reference group is always “poor people.”

The introduction to the task and each block is identical to Wave 1.

The four questions for each block are then presented in a fixed order. The first two questions are new additions for this wave of data collection: “Do you think all [reference group] are basically the same – for example, they behave in the same way and like the same things?” (*reference group homogeneity*); and “Do you think all [non-reference group] are basically the same – for example, they behave in the same way and like the same things?” (*non-reference group homogeneity*).

The next item is repeated from the Wave 1 version of this task: “Is it possible for [non-reference group] to become [reference group]?” (*transformation*), If a child says “yes” or “maybe”, they are asked the following two follow-up questions (which were not included in Wave 1): “How?” and “Would something in the person need to change or would something in the world need to change?” If a child says “no” to this question, they are instead asked, “Why not?” and “Is it because of something in the person themselves or is it because of something in the world?” For both of these follow-up questions, the two response options (“something in the person” versus “something in the world”) are presented in a random order across children, but in the same order across blocks of the task for each child.

The final item is a repeated question from the Wave 1 Social Essentialism task: “Are [non-reference group] people’s souls different from [reference group] people’s souls?” (*spiritual insides*).

When the child has answered all four questions in that block, the researcher proceeds directly to the next block of the task, beginning with the introduction of the next two social groups.

***Adaptation for samples in Lebanon:*** The final set of reference and non-reference religious groups featured in the Social Essentialism task for the five samples from Lebanon is presented in Table 3.

**Table 3:** Sample-specific item selection for the Social Essentialism task, for the six religions included in samples from Lebanon.

	Religion				
	*Druze faith*	*Maronite Christianity*	*Orthodox Christianity*	*Shia Islam*	*Sunni Islam*
**Reference group label (Religion Block)**					
plural noun form	Druze	Maronites	Orthodox	Shiites	Sunnis
	الدروز	الموارنة	الأورثوذوكس	الشيعة	السنة
adjective form	Druze	Maronite	Orthodox	Shiite	Sunni
	درزي	ماروني	أورثوذوكس	شيعي	السني
**Non-reference group label (Religion Block)**					
plural noun form	Christians	Orthodox	Maronites	Sunnis	Shiites
	المسيحي	الأورثوذوكس	الموارنة	السني	الشيعي
adjective form	Christian	Orthodox	Maronite	Sunni	Shiite
	المسيحي	الأورثوذوكس	الماروني	السني	الشيعي

***Planned analyses:*** For single-time point (cross-sectional) analyses, see the planned analyses in [1]. We note that, just as item-level analyses will be critical for interpreting response patterns in Wave 1, item-level analyses of Wave 2 will be particularly important for understanding both how children reason about social categories and how children interpret the particular questions posed to them in this version of the protocol.

Additional single-time point analyses will focus on children’s open-ended and forced-choice responses to follow-up questions regarding the *transformation* item (“Is it possible for [non-reference group] to become [reference group]?”). In particular, we plan to code children’s open-ended responses for spontaneous intrinsic versus extrinsic explanations related to why it was, or was not, possible to change one’s social category membership [44–47]. See [48] for a possible coding scheme.

Across the network, there is widespread interest in social essentialism as both a predictor of beliefs and behaviors and as an outcome of interest in its own right. Multi-time point analyses might include examinations of relationships between responses to the Wave 1 versus Wave 2 versions of the Social Essentialism task, as well as examinations of the degree to which individual-level changes in social essentialism across timepoints might explain changes in responses to other parts of our protocols. We note, however, that within-subjects analyses of individual items will only be possible for the subset of social groups included at both waves for any given cultural-religious sample, and to the two questions included in both versions of this task (*spiritual insides* and *transformation*). Likewise, any within-subjects analyses of composite social essentialism scores (combining responses across items) will need to take into account the differences in tasks across waves.

Further analyses might include assessments of the validity of each version of the Social Essentialism task in a given cultural-religious setting; comparisons across cultural-religious samples; and correlations between Social Essentialism and other tasks in the Child Protocol.

#### Norm Violations task.

***Rationale for retention and revision of task:*** The Wave 2 Child Protocol features a slightly modified version of the Norms Violation task from Wave 1. To reduce the overall length, the Wave 2 Child Protocol does not include the familiarity assessment portion of the Wave 1 Norm Violations task. The Wave 2 Child Protocol additionally no longer includes severity ratings for the questions about alterability and outgroup members, and the conventional norm used across samples is changed from the norm used in Wave 1. Including this task with only minor changes in both waves allows us to examine the development of children’s reasoning about violations of religious, moral, and conventional norms from a within-subjects perspective.

***Task design and procedure:*** The researcher provides the same introduction to this task as used in Wave 1 [see 1] and provides the child with two practice trials to familiarize children with the task; one practice trial focuses on an action that is clearly impermissible (insulting someone), and the other focuses on an action that is clearly permissible (giving someone a present). As in Wave 1, the gender of the characters featured in this task are matched to the participating child’s gender; if the participating child does not identify as a boy or a girl, or if the participating child’s gender is unknown, the child is shown the “woman” version of the task.

The task consists of four blocks of questions presented in a random order, each block centered on the violation of a particular norm (two religious norms, one moral norm, and one conventional norm; see Table 4 for norms selected for each sample in Lebanon).

**Table 4:** Sample-specific item selection for the Norm Violations task, for the five samples from Lebanon.

	Religion				
	*Druze faith*	*Maronite Christianity*	*Orthodox Christianity*	*Shia Islam*	*Sunni Islam*
**Religious Norm Violation #1**					
Item (woman version)	This is a woman named Rola. Rola is Druze. Rola eats pork.	This is a woman named Maria. Maria is Maronite. Maria doesn’t fast before Easter.	This is a woman named Maria. Maria is Orthodox. Maria doesn’t fast before Easter.	This is a woman named Fatima. Fatima is Shia. Fatima prays without doing wudu’ (ablution).	This is a woman named Fatima. Fatima is Sunni. Fatima prays without doing wudu’ (ablution).
	هيدي مرا إسما رولا. رولا درزية. و رولا بتاكل لحم الخنزير.	هيدي مرا إسما ماريا. ماريا مارونية. و ماريا ما بتصوم قبل عيد الفصح.	هيدي مرا إسما ماريا. ماريا أورثوذوكس. و ماريا ما بتصوم قبل عيد الفصح.	هيدي مرا إسما فاطِمَة. فاطِمَة فاطِمَة شيعية. و بتصلي بلا ما تتوضا	هيدي مرا إسما فاطِمَة. فاطِمَة سنية. و فاطِمَة بتصلي بلا ما تتوضا
Character name, religious group member (woman/man)	Rola/Wissam	Maria/George	Maria/George	Fatima/Ali	Fatima/Omar
	رولا\وسام	ماريا\جورج	ماريا\جورج	فاطِمَة\علي	فاطِمَة\عمر
Character name, religious group non-member (woman/man)	Christine/Tony	Fatima/Ali	Fatima/Ali	Maria/George	Maria/George
	كريستين\طوني	فاطِمَة\علي	فاطِمَة\علي	ماريا\جورج	ماريا\جورج
**Religious Norm Violation #2**					
Item (woman version)	This is a woman named Samar. Samar is Druze. Samar enters the khalweh from the men’s entrance.	This is a woman named Christine. Christine is Maronite. Christine never attends mass.	This is a woman named Christine. Christine is Orthodox. Christine never attends mass.	This is a woman named Zeinab. Zeinab is Shia. Zeinab eats food that isn’t halal.	This is a woman named Aya. Aya is Sunni. Aya eats food that isn’t halal.
	هيدي مرا إسما سمر. سمر درزية. و سمر بتفوت عالخلوة من مدخل الرجال.	هيدي مرا إسما كريستين. كريستين مارونية. و كريستين ما بتحضر قداس أبدًا.	. هيدي مرا إسما كريستين. كريستين أورثوذوكس. و كريستين ما بتحضر قداس أبدًا.	هيدي مرا إسما زينب. زينب سنية. وزينب بتاكل أكل مش حلال.	هيدي مرا إسما آية. آية سنية. و آية بتاكل أكل مش حلال.
Character name, religious group member (woman/man)	Samar/Kamel	Christine/Tony	Christine/Tony	Zeinab/Haidar	Aya/Khaled
	سمر\كامل	كريستين\طوني	كريستين\طوني	زينب\حيدر	آية\خالد
Character name, religious group non-member (woman/man)	Maria/George	Zeinab/Haidar	Zeinab/Haidar	Christine/Tony	Christine/Tony
	ماريا\جورج	زينب\حيدر	زينب\حيدر	كريستينطوني	كريستين\طوني
**Moral Norm Violation**					
Item (woman version)	This is a woman named Nadia. Nadia is Druze. Nadia hits other people for no reason.	This is a woman named Rosette. Rosette is Maronite. Rosette hits other people for no reason.	This is a woman named Rosette. Rosette is Orthodox. Rosette hits other people for no reason.	This is a woman named Batoul. Batoul is Shia. Batoul hits other people for no reason.	This is a woman named Hind. Hind is Sunni. Hind hits other people for no reason.
	. هيدي مرا إسما ناديا. ناديا درزية. و ناديا بتضرب ناس تانيين بلا سبب	هيدي مرا إسما روزيت. روزيت مارونية. و روزيت بتضرب ناس تانيين بلا سبب	هيدي مرا إسما روزيت. روزيت أورثوذوكس. و روزيت بتضرب ناس تانيين بلا سبب	هيدي مرا إسما بتول. بتول شيعية. و بتول بتضرب ناس تانيين بلا سبب	هيدي مرا إسما هند. هند سنية. و هند بتضرب ناس تانيين بلا سبب
Character name, religious group member (woman/man)	Nadia/Adham	Rosette/Joseph	Rosette/Joseph	Batoul/Jaafar	Hind/Walid
	ناديا\أدهم	روزيت\جوزيف	روزيت\جوزيف	بتول\جعفر	هند\وليد
Character name, religious group non-member (woman/man)	Rosette/Joseph	Batoul/Jaafar	Batoul/Jaafar	Rosette/Joseph	Rosette/Joseph
	روزيت\جوزيف	بتول\جعفر	بتول\جعفر	روزيت\جوزيف	روزيت\جوزيف
**Conventional Norm Violation**					
Item	This is a woman named Hiam. Hiam is Druze. Hiam wears pyjamas when leaving to go to work.	This is a woman named Claudia. Claudia is Maronite. Claudia wears pyjamas when leaving to go to work.	This is a woman named Claudia. Claudia is Orthodox. Claudia wears pyjamas when leaving to go to work.	This is a woman named Rabab. Rabab is Shia. Rabab wears pyjamas when leaving to go to work.	This is a woman named Amena. Amena is Sunni. Amena wears pyjamas when leaving to go to work.
	هيدي مرا إسما هيام. هيام درزية. و هيام بتلبس بيجاما هيي ورايحة عالشغل	هيدي مرا إسما كلوديا. كلوديا مارونية. و كلوديا بتلبس بيجاما هيي ورايحة عالشغل	هيدي مرا إسما كلوديا. كلوديا أورثوذوكس. و كلوديا بتلبس بيجاما هيي ورايحة عالشغل	هيدي مرا إسما رباب. رباب شيعية. و رباب بتلبس بيجاما هيي ورايحة عالشغل	هيدي مرا إسما آمنة. آمنة سنية. و آمنة بتلبس بيجاما هيي ورايحة عالشغل
Character name, religious group member (woman/man)	Hiam/Wael	Claudia/Michel	Claudia/Michel	Rabab/Hussein	Amena/Ahmad
	هيام\وائل	كلوديا\ميشال	كلوديا\ميشال	رباب\حسين	آمنة\أحمد
Character name, religious group non-member (woman/man)	Claudia/Michel	Rabab/Hussein	Rabab/Hussein	Claudia/Michel	Claudia/Michel
	كلوديا\ميشال	رباب\حسين	رباب\حسين	كلوديا\ميشال	كلوديا\ميشال
**Religious authority**					
	Allah	Allah	Allah	Allah	Allah
	الله	الله	الله	الله	الله

At the beginning of each block, the child is presented with a grayscale silhouette of character, described as an adult member of the target religious group who violates the norm. For religiously affiliated children, this adult is described as a member of their religious group; see further details about target religious groups under “Child as Transmission task,” above.

For each sample, the Wave 2 Child Protocol features the same two religious norms and the same moral norm used in Wave 1.

The conventional norm employed in Wave 1—wearing socks on one’s hands—was flagged by network members as having direct negative consequences for the person who engages in the norm violation (e.g., making it harder for them to grasp objects). In Wave 2, we replace this with a new, standardized conventional norm that better aligns with standard definitions of “conventions” in the psychological literature [49,50]: either “wears pajamas [to the office] to work” or “wears a swimsuit [to the office] to work” (the phrase “to the office” is included in some but not all adaptations of this task across the network). This decision emerges from an iterative process of development within and across research teams, with particular attention to the idea that following or breaking the norm should not have intrinsic or direct consequences for the welfare or goals of the person who violates the norm or the people around him or her; additional considerations include choosing a conventional norm that is widely followed by members of the local community, with which children are familiar, and which is not rooted in religion.

Within each block of questions, Wave 2 features the same fixed order questions used in Wave 1. The child first judges and explains the *permissibility* of a norm violation by a religious group member (e.g., “This is a woman named Fatima. Fatima is Shia. Fatima prays without doing wudu’ (ablution). Is that okay or not okay? Why is that okay/not okay?”). Then the child rates the *severity* of the norm violation (“And how good/bad is it that...? Not good/bad, just a little bit good/bad, or very good/bad?”) and assesses the *alterability* of the norm by a religious authority (e.g., “What if God said that it was okay to [violate norm] – then would it be okay...?”). Finally, the child is asked to judge the *permissibility* of the norm violation if it were committed by someone who is not a member of the religious group.

These questions are identical to Wave 1; the only modification is the omission of two severity ratings questions within each block (after the question about alterability by a religious authority, and after the question about an non-group member).

***Adaptation for samples in Lebanon:*** The final set of properties featured in the Norm Violations task for the five samples from Lebanon is presented in Table 4.

***Planned analyses:*** For single-time point (cross-sectional) analyses, see the planned analyses in [1]. Note that because the Wave 2 Child Protocol no longer includes severity ratings for the questions about alterability and outgroup members, analyses including these questions cannot be conducted with Wave 2 data.

Multi-time point analyses might include examinations of relationships between responses to the Wave 1 versus Wave 2 versions of the Norm Violations task, as well as examinations of the degree to which individual-level changes across timepoints might be related to responses to other parts of our protocols. We note that exact within-subjects comparisons will only be possible for the subset of norms included at both waves for any given cultural-religious sample. We acknowledge that, although this is not the ideal approach for formal longitudinal analyses, if the Wave 2 conventional norm functions similarly to that in Wave 1–a reasonable assumption in the absence of theoretical reasons to expect otherwise– it may still be conceptually informative to include both waves of conventional norms in analyses when examining patterns of norm violation judgments. Likewise, any within-subjects analyses of composite scores derived from this task (i.e., attempts to combine responses across questions) will need to take into account the difference in conventional norm across waves.

Further analyses might include comparisons across cultural-religious samples, focusing on how religious affiliation versus other social-cultural factors might shed light on any observed similarities and differences across samples for other tasks in the Child Protocol.

#### Direct questions about religion.

***Rationale for retention and revision of task:*** The final element of the Wave 2 Child Protocol is a set of questions designed to directly assess children’s explicit understanding of religion and their own religious identity [see, e.g., 51–53]. Some of these questions are retained without changes from Wave 1; the purpose of including similar questions in both waves of data collection is to observe individual differences in children’s understanding of religion and their own identity. To further explore the development of religious identity, two new sets of questions are included in the Wave 2 Child Protocol; one focuses on the child’s definition and identity of the child’s religious ingroup, and the other focuses on the child’s awareness of and participation in religious rituals.

***Task design and procedure:*** The child is presented with the following set of questions in a fixed order.

First, the researcher asks three open-ended questions about religion, taken directly from Wave 1: 1) “When I say the word ‘religion,’ what do you think of? Can you explain what ‘religion’ means?”; 2) “There are lots of different religions in the world. Can you tell me all the religions you’ve heard of?”; and 3) “What about your family: Does your family belong to a certain religion?” [or, in some cases, “Does your family believe in a certain religion?”; see 1].

Then the researcher proceeds to a series of new questions, not included in Wave 1. The first set of questions all focus on a religious “ingroup” relevant to the child (e.g., “Maronite” for Maronite Catholic children). For sites that include religiously unaffiliated children (e.g., England, the United States), their “ingroup” for this question is “not religious.” The researcher begins by saying, “Okay. I know some people around here are [religious ingroup, e.g., ‘Maronite’]” and asks the following three questions [based on 51]: “What does it mean to be [religious ingroup]?”; “How do you know if someone is [religious ingroup]?”; and “How does a person become [religious ingroup]?”

The second set of questions focus on one important or salient religious ritual relevant to either the child’s own religious ingroup or to a prominent religious group in the child’s local setting. The researcher introduces this ritual by saying, “I’ve heard that one thing that [religious ingroup, e.g., ‘Maronite’] people do is [perform religious ritual, e.g., ‘take first communion’]. What does it mean to [perform religious ritual]?” If the child is unfamiliar with the ritual, the researcher does not ask any further questions. If the child provides a response, regardless of accuracy, they are asked four additional questions about the ritual: 1) “What kinds of things do people do when they [perform religious ritual]?”; 2) “Why do they [perform religious ritual] that way?”; 3) “Could someone [perform religious ritual] in a different way?”; and 4) “Have you ever [performed religious ritual]?” Religious rituals vary across sites and samples. Research teams are instructed to select a ritual that would be familiar to children, and, where possible, to select a ritual that is in some way related to growing up or becoming a more full-fledged member of the religious group (e.g., a coming of age ritual or initiation). Priority is given to rituals that children participate in themselves or observe at a certain age.

As in Wave 1, at the end of the interview the child is asked, “Is there anything else you want me to know about you and your family?”

***Adaptation for samples in Lebanon:*** The final set of religious rituals featured in the Wave 2 Direct Questions about Religion task for the five samples from Lebanon is presented in Table 5.

**Table 5:** Sample-specific item selection for the Direct Questions about Religion task, for the five samples from Lebanon. See main text for descriptions of network-wide guidelines for item selection.

Religion				
*Druze faith*	*Maronite Christianity*	*Orthodox Christianity*	*Shia Islam*	*Sunni Islam*
I’ve heard that one thing that Druze people request entry into the faith.	I’ve heard that one thing that Maronite people do is take first communion.	I’ve heard that one thing that Orthodox people do is get baptized.	I’ve heard that one thing that Shia people do is pray.	I’ve heard that one thing that Sunnah people do is pray.
سمعت إنو وحدة من الإشيا لي بيعملوها الناس الدروز إنو بيطلبوا دينن	سمعت إنو وحدة من الإشيا لي بيعملوها الناس الموارنة إنو بيعملوا أول قربانة	سمعت إنو وحدة من الإشيا لي بيعملوها الناس الأورثوذوكس (يعني روم أورثوذوكس) إنو بيتعمدوا	سمعت شي عن الناس الشيعة إنو بصلوا	سمعت شي عن الناس السنة إنو بصلوا

***Planned analyses:*** Analyses of the open-ended questions retained from Wave 1 (e.g., “When I say the word ‘religion,’ what do you think of? Can you explain what ‘religion’ means?”) will be qualitative, using the themes and codes developed from children’s responses in Wave 1 (not yet developed as of the submission of this manuscript).

We anticipate researchers will apply both cross-sectional and within-subjects analysis techniques to the data from the questions that have been retained across waves. Cross-sectional analyses include comparisons of samples from different cultural-religious groups, to further our understanding how children define religion and come to self-identify as members of religious groups. Single-timepoint (cross-sectional) analyses include individual differences in children’s understanding of what religion is, their awareness of specific religions, and their own sense of religious identity.

For the new questions added to the Wave 2 protocol, we predict that children who identify themselves as part of a religious ingroup will provide richer and more “accurate” (i.e., more aligned to the cultural setting) responses to questions about the religious ingroup and the group-relevant religious ritual. Analyses of these hypotheses will be cross-sectional.

### Parent/Caregiver Survey

At each timepoint, parents (or other caregivers/guardians) are asked to complete a survey to help us better understand mechanisms underlying the transmission of religious information from parents/caregivers to their children, as well as broader social and cultural influences on children’s religious development. The primary goal of the Wave 2 Parent/Caregiver Survey is to help us understand any significant changes in the child’s religious exposure and experience that may have occurred between Wave 1 and Wave 2. As such, the survey is significantly shorter than in Wave 1, consisting of two required components and one optional component (described below). Unlike the Child Protocol, the Wave 2 Parent/Caregiver Survey does not vary by sample within a fieldsite (e.g., in Lebanon, a Maronite Christian parent sees the same survey as an Orthodox Christian parent).

Parents/caregivers are permitted to skip any question they prefer not to answer. Parents/caregivers with more than one child participating in the study are asked to complete one survey per child.

#### Demographics and Child Religion.

***Task design and procedure:*** In Wave 2, parents/caregivers are asked to indicate their relationship to the child (as in Wave 1). Then they are asked a short series of questions to update the demographic and religious information collected about their child during Wave 1, including the child’s current age and current grade in school, as well as the child’s educational experiences to date; parents/caregivers indicate whether the child has ever been homeschooled (religiously or secularly), has ever attended a secular or religious school, or has ever participated in other forms of religious or spiritual education. Parents/caregivers are also asked to indicate the frequency of their child’s involvement in religious and spiritual activities and practices over the past year, as well as the frequency with which their child attended religious services over the past year.

***Adaptation for samples in Lebanon:*** This task does not require any sample-specific customization, beyond translation.

***Planned analyses:*** As our focus in this paper is to lay out the methods and analyses for the Wave 2 Child Protocol, we do not detail planned analyses for the Parent/Caregiver Survey here.

#### Parental Ethnotheories Measures (optional).

***Task design and procedure:*** Research teams are given the option to include a Parental Ethnotheories measure in the Wave 2 Parent/Caregiver Survey, adapted from the measure developed in Wave 1, which was designed to document parents’/caregivers’ beliefs about how, where, and from whom children should learn about religion and social issues [1]. The Wave 2 adaptation uses the same questions, adapted to document beliefs about how, where, and from whom children should learn about medical and health-related issues. Given the timing of the administration of Wave 2 in relation to the COVID-19 pandemic, parents/caregivers are explicitly asked to respond to the questions as though there were no restrictions related to COVID in place.

Parents/caregivers are first asked to rate their agreement with 14 statements reflecting beliefs about how, where, and from whom children should learn about medical and health-related issues (e.g., “It is important for my child to read and understand books about medical and health-related issues”; “My child should ask questions about medical and health-related issues.”). Questions are presented in a random order and parents/caregivers respond on an 11-point scale, from “I do not agree with this at all” to “I completely agree with this.”

Some of these questions feature follow-up questions about ethnotheories of development in the medical and health-related domain; for example, after rating their agreement with the statement “My child should only learn about medical- and health-related issues in our home,” parents/caregivers are then asked to select any other locations where it would be okay for children to learn about these issues (e.g., at school, at their friend’s house, at a religious site, through a TV show).

After completing this set of questions, parents/caregivers are asked to select the age at which they believe children *should* know about medical and health-related issues (with ages provided in two-year increments from 0–2 years to 18 years and older; “never” is also included as an option); to report the age at which they believe children tend to start asking questions about medical and health related issues (with the same response options); and to describe the specific medical or health-related issues they had in mind when answering these questions (open-ended). Parents/caregivers are also given the opportunity to provide any additional information about their views.

***Adaptation for samples in Lebanon:*** This task does not require any sample-specific customization, beyond translation.

***Planned analyses:*** As our focus in this paper is to lay out the methods and analyses for the Wave 2 Child Protocol, we do not detail the planned analyses for the Parent/Caregiver Survey here.

#### Open-ended parent questions.

The following section of the Wave 2 Parent/Caregiver Survey include a set of open-ended questions to obtain additional information about the parent/caregiver’s and child’s experiences with religion since the first time they participated.

***Task design and procedure:*** The Wave 2 Parent/Caregiver Survey includes a set of questions designed to give parents/caregivers an opportunity to reflect more freely on their child’s religious development (and, if included in the sample, on their own ethnotheories about religion and medical and health-related issues). Research teams are given the option to include these open-ended questions at the end of their parent/caregiver survey for parents to complete on their own or as a qualitative oral interview done with a researcher where responses are recorded and transcribed.

The first three questions ask parents/caregivers to reflect on what might have changed since the previous wave of data collection: 1) “Some time has passed since you first participated in this study. Has anything changed in how your child learns about religion?”; 2) “Now let’s talk about religious activities your child participates in. Has anything changed about the activities related to religion that your child is involved in in school, at home or in the community, more generally?”; and 3) “Now let’s talk about you. Has anything changed in the ways in which you talk, teach, or answer questions about religion with your child?” In all cases, parents are asked to describe these changes.

Parents/caregivers are then asked to describe whether they attribute changes in their approach to their child’s religious development to a number of specific factors, including: “My own views about what to discuss about religion with my child have changed”; “My own religious beliefs have changed”; “Participating in this study last year had an impact on how we approach religion in our home (e.g., child has been asking more questions about religion; my child has been more interested in participating in religious activities)”; “My child is older now so new approaches are needed”; “The COVID-19 situation has changed since last year and this has had an impact on my family’s approach to religion.” Parents/caregivers are also given the option to report some other reason for changes in their approach, to report no changes in their approach, or to decline to respond to this question.

The next two questions ask parents/caregivers to reflect on the specific material and social sources that their child might use to learn about religion.

First, parents/caregivers are asked, “In many places, children learn about religion through prayers, books, stories, songs, videos, or other activities. Thinking about your child, please tell us about one or two examples that your child has been exposed to at home or at school that they spend a lot of time with or engage with regularly. Is there a particular book, story, song, video, or activity that you like for helping teach your child about religion in general or the beliefs and practices that are valued in your family? What makes you like it? How do you think this has taught your child about these ideas and values?” In addition to eliciting reflections on the specific circumstances of children in this study, this question is designed to lay the foundation for compiling a corpus of religious materials for future analysis.

Second, parents/caregivers are asked, “Thinking about your child, is there a particular person or group of people who have impacted your child’s learning about religion?”

Finally, for any samples that opts to include the Parental Ethnotheories measure (described in the previous section), a final open-ended question asks parents/caregivers to describe their feelings about when and how children should learn about medical- and health-related issues.

***Planned analyses:*** As our focus in this paper is to lay out the methods and analyses for the Wave 2 Child Protocol, we do not detail the planned analyses for the Parent/Caregiver Survey here.

### Other information

The following sections have been adapted from the DBN Wave 1 Study Protocol [1].

#### Data management plans.

Data from the Child Protocol and Parent/Caregiver Survey are collected through Qualtrics survey software. When authorized, audio and/or video recordings of the child interview are collected either using a portable recording device or using tele-conferencing software (e.g., Zoom, Microsoft Teams). These recordings are used to help transcribe responses to open-ended questions in the child interview.

Once datasets have been collected, data cleaning will be performed by each team. Data from participants who authorize that their de-identified responses be shared publicly will be shared on platforms for open- science, including the Open Science Framework (https://osf.io/). Likewise, audio/video recordings from participants who authorize sharing these recordings will be shared with the research community on Databrary (https://www.databrary.org/).

Once all datasets from the second wave of data collection have been cleaned and de-identified, the current members of the DBN will have exclusive access to the full dataset for a finite interval of time. Following that interval, the data will then be shared publicly as described above.

#### Ethical considerations and declarations.

This study has been approved by the Institutional Review Board for Socio-Behavioral research (IRB-SB) at the University of California, Riverside, under protocol #HS-21–124;, and by the Institutional Review Board (Charles River IRB Office) at Boston University under protocol #4631E. Together, these IRBs cover data collection for all US samples as well as the samples in Mexico and Taiwan R.O.C. Data collection in Lebanon has been ethically reviewed and approved by the Institutional Review Board for Social and Behavioral Sciences at the American University of Beirut, Beirut, Lebanon under protocol #SBS-2021–0343. Parents/caregivers give informed consent either in writing (on paper or via an online survey) or verbally via a live conversation with the researcher (in person or over video chat); and children give oral assent. The mode of consent varies across these samples depending on the modality of participation (online vs. in person) and on the degree to which parents/caregivers are comfortable reading written text. Additional information regarding the ethical, cultural, and scientific considerations specific to inclusivity in global research is included in the Supporting Information.

All other planned samples are covered by ethics boards at the home institutions of their respective research team leaders (who are independent subaward PIs). Research team leaders are also responsible for securing approval from any additional community organizations, school boards, or governmental bodies overseeing research in their field sites and samples.

#### Status and timeline.

As this is the second wave of a multi-time point study, recruitment for the study is complete in all of the samples that completed Wave 1 of this study, although we anticipate continuing to add samples to this data collection effort in the future. All participants are contacted nine to 15 months after their first participation at Wave 1 to participate again at Wave 2. At the time of this submission, data collection is underway in 40 cultural-religious samples, and is complete in 21 of these samples. One additional sample is set to begin data collection in Summer 2025. We anticipate data collection for all of the 41 samples described in the current manuscript to be complete by January 31, 2026. We expect to share results from this study approximately 18 months after data collection is complete.

## Discussion

This manuscript presents a study protocol designed for 5- to 13-year-old children and their parents/caregivers across 41 cultural-religious samples, for the second wave of data collection in a large, multi-time point study of the development and diversity of religious cognition. The study protocol is designed to repeat, modify, and extend the methods devised by the Developing Belief Network (DBN) for our Wave 1 Study Protocol [1], and reflects an additional seven months of intensive, iterative collaboration involving over 50 research team leaders, with input from many additional local research team members. The result is a study protocol that the DBN believes is well-suited to describe key aspects of religious conceptual development across diverse cultural-religious contexts. In particular, it is designed to address the three main research questions of the DBN: How do children represent and reason about religious and supernatural agents? How do children represent and reason about religion as an aspect of social identity? And how are religious and supernatural beliefs transmitted within and between generations?

A core mission of the DBN is to integrate open science principles into our methods and analyses. The goal of this manuscript is to enable future researchers to make use of the rich datasets generated by this collaboration and to provide materials so that adaptations of the current protocol can be used with new sites and samples. With this in mind, we first provide guidance for how future research teams might effectively implement DBN protocols with new sites and samples. We then offer our reflections on the benefits and challenges of developing and implementing large-scale, multi-site, multi-time point studies; how our network navigated such challenges; and our suggestions for how future researchers might learn from our experiences and build on the work presented here.

For researchers hoping to implement DBN protocols to study religious conceptual development with new sites and samples, we recommend using the Wave 2 Child Protocol described in this manuscript, together with the Wave 1 Parent Survey and Caregiver-Child Conversation task detailed in [1]. As noted above, this is the approach we implement for the new cultural-religious samples that have joined the network since the launch of Wave 2. The Wave 2 Child Protocol is substantially shorter (estimated time to complete: 40–50 minutes, compared to ≥ 90 minutes for the Wave 1 Child Protocol), and includes refinements to improve children’s understanding and experience of the study tasks, and to increase researchers’ abilities to interpret children’s responses. The Wave 1 Parent Survey provides more detailed information about children’s upbringing, material circumstances, and educational experiences; as well as parents/caregivers’ orientation toward religion and child-rearing. In the current multi-time point study it is unnecessary (and cost-prohibitive) to ask parents/caregivers to provide this information a second time, but for new sites and samples we would highly recommend this. Finally, most network members report that the Caregiver-Child Conversation task (from Wave 1) has been well-received by participating families in nearly all sites and samples; it is not repeated at Wave 2 for practical reasons (namely, parents’ time and availability), but we would highly recommend this task for other research groups.

### Limitations

Throughout the manuscript, we discuss how the modifications to the child protocol between Waves 1 and 2 will enhance versus complicate our own efforts to conduct within-subjects analyses for the children included in the current multi-time point project (see, e.g., the “Planned analysis” sections for each task). Here, we focus instead on what new research teams should consider in implementing and adapting the Wave 2 Child Protocol presented in the current manuscript on its own (for example, with new sites and samples).

First, many of the researchers in the DBN—especially those collecting data with children who have had less exposure to Western-style education or one-on-one conversations with adults—would caution new research teams that this protocol is still quite long for children younger than 7 years of age. Researchers implementing this protocol would be wise to conduct thorough piloting to determine an appropriate starting age for their sample. This might yield a modified protocol, limited to a subset of the tasks likely to be most appropriate for younger children within a given sample and for which younger children’s performance offers a meaningful point of comparison for older children.

Research groups with a particular interest in one of the tasks in this protocol might also consider reverting to the Wave 1 version of that task, at least for older children. In particular, the Memory Span, Property Attributions, Child as Transmission Agent, and Norm Violations tasks were reduced in length by simply cutting questions from the Wave 1 versions (rather than by introducing major modifications to the design of the task). In these cases, these cuts are motivated almost entirely by concerns about the length of the overall study protocol, rather than concerns about the Wave 1 tasks themselves. New research groups might consider whether it is more prudent to preserve these cuts or instead reinstate the full-length versions included in the Wave 1 protocol.

### Reflections on multi-site, multi-time point research

We now reflect on the challenges of developing a study protocol for our second wave of data collection while the first wave of data collection was still ongoing in some sites. Although these reflections are rooted in our experience of studying the development of religious cognition, we consider them relevant for any large-scale, multi-site, multi-time point study involving human participants, especially if the work involves children, understudied populations, new or under-resourced field sites, or participants living in precarious situations (e.g., health crises; social, political, and economic crises; natural disasters)—conditions which are all relevant to the network of field sites included in the current project.

As any researcher working across cultural settings or across multiple time points would stress, multi-site and multi-time point studies require a significant amount of resources. This five-year study was fortunate enough to be funded by a $10 million grant from the John Templeton Foundation, enabling us to embark on such an ambitious project. Time was a more difficult resource to budget. When the network was designed, we allocated one year for study design, followed by four years in which to complete three waves of data collection. In practice, this timeline has been challenging, both in the initial phase of designing and piloting methods for our first wave of data collection, and in the rapid turn-around between Wave 1 and Wave 2.

First, we faced significant challenges piloting our methods across the full range of ages, religious groups, and cultural settings represented by the network prior to launching data collection for Wave 1. In part, these challenges were a result of the COVID-19 pandemic, which began directly before the launch of the DBN (Fall 2020) and has continued to impact social interactions throughout both waves of data collection. Although the pandemic might appear to some readers to be a unique obstacle to research, our experiences in the current project instead highlight how common it is for research outside of the US and other “WEIRD” [12] or “Minority World” [13] settings to be conducted during extremely disruptive events and circumstances. During the first year of the project—the year we had set aside for the collaborative design and iterative refinement of our study protocol—individuals living and working in all of the field sites represented in our network faced a variety of effects of the global pandemic, including high death rates in a few field sites, severe travel restrictions in many field sites, and the suspension of in-person schooling in nearly all field sites. However, the pandemic was not the only obstacle we faced in that first year. Research teams and participating families also lived through major economic collapses (e.g., in Lebanon); major political crises (e.g., in Peru and the US); severe flooding, cyclones, earthquakes, and other natural disasters (e.g., in England, India, Indonesia, Mexico, and Uganda); as well as wars and other forms of ongoing and escalating violence that directly affected many of our researchers and participating families (e.g., Jewish and Palestinian Muslim families in Israel, as well as families in Lebanon, Mexico, and the US). Clearly, such events resulted in enormous impacts not only on the day-to-day logistics of conducting research, but also on the availability, interest, and needs of both researchers and potential participants—not to mention their physical safety and general well-being. 2020–2021 may have been a particularly difficult time to launch a project of this size, but we would urge other researchers not to discount this experience as exceptional. Conducting research in this many field sites, and in field sites this diverse, involves taking on the risk and responsibility of working under such conditions; this, in turn, confers tremendous logistical challenges and ethical responsibilities onto each research team and onto the collaborative more generally. In practical terms, we strongly suggest budgeting more than one year for study design and piloting prior to the launch of such an ambitious multi-site study.

By the same token, our planned timeline for data collection, in which Wave 2 was intended to launch approximately one year after the launch of Wave 1, proved to be very tight. Allotting more time for piloting prior to launching Wave 1 might have allowed us to refine and adapt the protocol for the full range of field sites and samples represented in our network; this, in turn, might have allowed us to make some of the improvements to the protocol documented in the current manuscript in time for Wave 1 data collection, rather than Wave 2. However, even with more extensive piloting prior to Wave 1, we would also suggest that future research teams budget more time for data cleaning, data analysis, collaborative discussion, and refinement of methods between waves of data collection. Although approximately one year of data collection was enough time for most research teams to begin collecting Wave 1 data and report back on their experiences of implementing the study in their field sites, it was not enough time for *all* cultural-religious samples to contribute equally to this refinement process. Only a few research teams had completed Wave 1 data collection by the time we needed to develop Wave 2, and some had yet to launch Wave 1 in all cultural-religious samples. Likewise, many researchers in the network shared the impulse to make data-driven decisions about necessary modifications to the protocol, or interesting ways to follow up on the results of our first wave of data collection—but we also shared a strong desire to avoid engaging in significant methodological changes based on the limited subset of completed Wave 1 samples. We feared such decisions would be biased toward the needs, interests, and responses of participants for whom participation was most convenient and familiar. In an ideal world, for a project of this magnitude [and this style of collaboration; see 2] we would suggest budgeting more time for data analysis, iterative redesign, and additional piloting between waves of data collection.

## Conclusion

In conclusion, the study protocol presented here is not without its limitations, but we believe that these tasks will surface unique insights into the ways in which children in a diverse range of cultural and religious settings come to think, behave, and experience the world around them. It is our intention that data and materials from Wave 1 and Wave 2 of this project, along with data from future waves of this collaborative research effort, will shed light on the development and diversity of religious cognition in early and middle childhood—a fundamental and still understudied aspect of conceptual development for the majority of children around the world. We hope that our reflections on the benefits and challenges of multi-site, multi-time point, developmental psychology research—as well as our descriptions of how this collaborative network navigated this challenges, and our suggestions for future researchers—will inspire others to continue this work and help deepen our understanding of the complexities of human experience and development.

## Supporting information

S1 Text Inclusivity in global research questionnaire. (DOCX)
