# Supplementary material for: Working across religions, cultures, settings, and development: Protocol for wave 2 data collection with children and parents by the developing belief network
Source: PLoS One. 2025 Sep 5;20(9):e0330727. doi: 10.1371/journal.pone.0330727 (PMC12412925; doi:10.1371/journal.pone.0330727)
Supplement: S1 Text — (DOCX) [file pone.0330727.s001.docx]

Inclusivity in global research

PLOS’ policy on inclusivity in global research aims to improve transparency in the reporting of research performed outside of researchers’ own country or community and ensures that PLOS publications reporting global research adhere to high standards for research ethics and authorship. Authors of relevant research articles may be asked to complete the questionnaire below, which outlines ethical, cultural, and scientific considerations specific to inclusivity in global research. This questionnaire may be requested when researchers have travelled to a different country to conduct research, if research uses samples collected in another country, research with Indigenous populations or their lands, or if research is on cultural artefacts. Researchers travelling to another country solely to use laboratory equipment will not normally be required to complete the questionnaire. However, the questionnaire can be requested at the journal’s discretion for any submission – if you have been requested to complete this questionnaire by the PLOS journal you submitted to, please do so.

Please complete the questionnaire below and include this as a Supporting Information file with your manuscript. Note that if your paper is accepted for publication, this checklist will be published with your article in the supporting information files. Please ensure that you reference the checklist in the main body of your manuscript. We suggest adding a subsection ‘Inclusivity in global research’ to your Methods section and adding the following sentence: “Additional information regarding the ethical, cultural, and scientific considerations specific to inclusivity in global research is included in the Supporting Information (SX Checklist)”

The questions have been designed to be applicable to a wide range of study types, and there are subsections for both human subjects research and non-human subjects research. If any of the questions are not relevant to your research please mark them as “N/A” as appropriate.

**Ethical considerations, permits and authorship**

*This section is applicable to all research types.*

Provide details as to who granted permissions and/or consent for the study to take place in the Methods section of your manuscript. This should include the names of **all** ethics boards, governmental organizations, community leaders or other bodies that provided approval for the study. If individuals provided approval refer to these people by their role or title but do not list their name(s).

Reported on page number: 65

If there were any deviations from the study protocol after approval was obtained please provide details of these changes in the Methods section of your manuscript.
Did this study involve local collaborators that are residents of the country where the research was conducted or members of the community studied? If you do not have any authors from said communities, please provide an explanation for this below.

Reported on page number: N/A

The PI has lived and worked (including conducting research) in Lebanon for over 20 years. The co-PI is of Lebanese descent, has lived in, conducted research, and has family in Lebanon. All the research team members were either Lebanese nationals or had grown up and/or studied in Lebanon. Research team members are affiliated with a variety of different religious groups. Therefore, our interest in conducting data collection in Lebanon in the context of the DBN emerged from our existing connection and interest in its communities.

Everyone listed as an author should meet PLOS’ criteria for authorship and all individuals who meet these criteria should be included in the author byline, rather than the acknowledgements. For further information please see the journal’s Authorship Policy.

**Human subjects research (e.g. health research, medical research, cross-cultural psychology)**

Did you obtain written informed consent from a representative of the local community or region before the research took place? How did you establish who speaks for the community? Details of written informed consent obtained from study participants should be reported separately in the Methods section of your manuscript.

Data collection in Lebanon was ethically reviewed and approved by the Institutional Review Board for Social and Behavioral Sciences at the American University of Beirut, Beirut, Lebanon (protocol # SBS-2021-0343.) See the following question regarding contact with community leaders.

How did members of the local community provide input on the aims of the research investigation, its methodology, and its anticipated outcome(s)?

Prior to Wave 1, we conducted semi-structured interviews with experts in the various religious belief systems relevant for our study. 12 interviews were conducted initially including priests, heads of religious instruction in schools, adults who were active in their particular religious community (currently or in their youth) and organized religious activities for children and were familiar with what children are exposed to. The research team was also diverse in terms of religious affiliation and so collectively the team was quite knowledgeable about the different belief systems and practices of most of the religious communities relevant to this study. Pilot interviews were also helpful in gauging children’s familiarity with various entities referred to in the protocol and how they are labeled.

When engaging with the local community, how did you ensure that the informed consent documents and other materials could be understood by local stakeholders?

One translator was working with us throughout the project and this person was also the research team coordinator and assisted with data collection and training of other research team members. Hence, she was intimately familiar with the project and contributed to the site-specific adaptations that were incorporated into the child protocol and surveys. She always prepared the initial translation of any protocol, presented it to the PI and Co-PI, they then reviewed the translation, reconsidered it alongside the English version and discussed any issues as a team (sometimes with additional research team members). The purpose of specific task items were also kept in mind during these discussions. For the materials addressed to Lebanese children (the consent form and the interview protocol), we chose to use colloquial Lebanese Arabic rather than formal Arabic. This decision was aimed at making the content more accessible for children, but it required careful consideration, especially for religious concepts. Materials addressed to Lebanese adults were in Modern Standard Arabic (the language of literacy). Our translator’s role extended beyond basic translation to include researching how these concepts are named in the religious community in question and simplified for children within Lebanon’s diverse religious landscape. This process included consultations with individual active in the various religious communities to confirm appropriate terminology, checking whether and how terms were typically simplified in the Lebanese dialect compared to Modern Standard Arabic or adult language. This collaborative approach helped ensure that the protocol was both comprehensible for children,culturally respectful and sociolinguistically appropriate (i.e. using the colloquial dialect or Modern Standard Arabic in appropriate ways).

Will the findings of the research be made available in an understandable format to stakeholders in the community where the study was conducted (e.g. via a presentation, summary report, copies of publications, etc.)? Please provide details of how this will be achieved.

Yes, the findings will be made available to stakeholders in the various Lebanese communities where the study will be conducted. In addition to providing copies of publications, we plan to prepare less technical summaries of our results. We will share these with the religious community leaders we met with who expressed interest in learning more about our study and its findings. In addition, we will share results with those responsible for religious education in the schools in the communities were data collection will be conducted. We also intend to report our results in other formats, such as local events (e.g., conferences, panels and workshops) on religious and social studies education. Of course, all results will be shared only in aggregate and following the IRB approved dissemination protocols.

**Non-human subjects research using specimens/ animals collected as part of the study, or those housed in archival collections. Examples include archaeology, paleontology, botany and zoology.**

Did the permission you obtained from a local authority to perform the study include an agreement on access to outputs and benefit sharing? This may include procedures to enable fair distribution of the benefits and resources arising from the research performed. Please include any details of Prior Informed Consent and Benefit Sharing Agreements obtained. These may be required by field-specific regulations, for example the Convention on Biological Diversity (CBD) and the associated Nagoya Protocol.

If the material used in your study was imported, please A) provide the year it was imported and B) indicate whether permits were obtained to import/export the materials used, C) provide details of any permits obtained. If this information is not available, please indicate this.

If you used archival specimens, please state how the material used in your study was acquired by the institute it is held in and provide details of any permits obtained for the original excavations/ sample collection. If this information is not available, please indicate this.

How was the potential cultural significance of the materials collected in your study to local communities considered in your research design? Were Indigenous peoples and/or local researchers and institutions involved with archaeological excavations / collection of specimens? If so, please provide a description of their involvement.

If your manuscript includes photographs of human remains please indicate whether authors obtained permission from descendants or affiliated cultural communities to do so.
